# Metallacages with 2,6-dipicolinoylbis(*N,N*-dialkylthioureas) as novel platforms in nuclear medicine for ^68^Ga, ^177^Lu and ^198^Au

**DOI:** 10.1186/s41181-023-00225-z

**Published:** 2023-11-20

**Authors:** Anna Baitullina, Guilhem Claude, Suelen F. Sucena, Eda Nisli, Cedric Scholz, Punita Bhardwaj, Holger Amthauer, Winfried Brenner, Christopher Geppert, Christian Gorges, Ulrich Abram, Pedro Ivo da Silva Maia, Sarah Spreckelmeyer

**Affiliations:** 1 Institute of Chemistry and Biochemistry, Freie Universität Berlin, Fabeckstr. 34-36, 14195 Berlin, Germany; 2 Charité - Universitätsmedizin Berlin, Corporate Member of Freie Universität Berlin, Humboldt-Universität zu Berlin, and Berlin Institute of Health, Department of Nuclear Medicine, Augustenburger Platz 1, 13353 Berlin, Germany; 3 Forschungsreaktor TRIGA Mainz, Johannes Gutenberg-Universität Mainz, Fritz-Strassmann-Weg 2, 55128 Mainz, Germany; 4 Núcleo de Desenvolvimento de Compostos Bioativos (NDCBio), Universidade Federal do Triângulo Mineiro, Uberaba, MG 38025-440 Brazil

**Keywords:** Metal complexes, Radiopharmaceuticals, Heterometallic complexes, Cancer, Metals in medicine

## Abstract

**Background:**

Heterometallic gold metallacages are of great interest for the incorporation of several cations. Especially in nuclear medicine, those metallacages can serve as a platform for radionuclides relevant for imaging or therapy (e.g. ^68^Ga or ^177^Lu). Moreover, the radionuclide ^198^Au is an attractive beta emitter, for potential application in nuclear medicine. Here, we aim to synthesize a new set of gold metallacages and to study their ability to coordinate to ^68^Ga, ^177^Lu and ^198^Au.

**Results:**

New heterometallic gold metallacages of composition [M{Au(L^morph^-κS)}_3_] (M = La^3+^, Tb^3+^, Lu^3+^ or Y^3+^) and [Ga{Au(L^morph^-κS)}_2_]NO_3_ have been synthesized from 2,6-dipicolinoylbis(*N,N*-morpholinylthiourea) (H_2_L^morph^) with [AuCl(THT)] and the target M^3+^ metal ions in yields ranging from 33 (Lu) to 62% (Tb). The characterization of the compounds bases on ESI–MS, ^1^H NMR, IR, EA and single-crystal X-ray diffraction techniques (all except the Ga derivative). Selected gold cages derived from H_2_L^morph^ were compared to previously reported gold cages that were derived from 2,6-dipicolinoylbis(*N,N*-diethylthiourea) (H_2_L^diethyl^). The tested metallacages show similar IC_50_ values close to that of auranofin in four different cancer cell lines (MCF-7, PC-3, U383, U343), e.g. 4.5 ± 0.7 µM for [Ga{Au(L^diethyl^)}_2_]NO_3_ on PC-3. The radiolabeling experiments thereof show high radiochemical purities with ^68^Ga and ^198^Au and low radiochemical purity with ^177^Lu.

**Conclusions:**

The results indicate that these gold metallacages could serve as a novel platform for inclusion of different (radio)nuclides with potential theranostic applications in nuclear medicine.

**Supplementary Information:**

The online version contains supplementary material available at 10.1186/s41181-023-00225-z.

## Background

Radiopharmaceuticals are radioactive compounds containing a radionuclide which can be used either for diagnostic, in the case of gamma (*γ*) or positron (*β*^+^) emitting radioisotopes, or therapeutic purposes, in the case of particle emitting radionuclides (*α*, *β*^−^ or Auger electron) (Lever et al. 2003; Reichert et al. 1999). The predominant isotope in diagnostic imaging is ^99m^Tc, due to its ideal nuclear properties (E_γ_ = 140 keV, T_1/2_ = 6.02 h), covering about 80% of worldwide clinical analyses using the SPECT (Single Photon Emission Computed Tomography) technique (Dilworth et al. 2015; Abram and Alberto 2006; Gielen and Tiekink 2005). In the past decades, the research in this field has been expanded to ^68^Ga ((t_1/2_ = 67.71 min, β^+^ 89%, E_β+ max_ = 1.9 MeV; EC 11%, E_γ max_ = 4.0 MeV), which has clinical utility due to the feasibility of the ^68^Ge/^68^Ga generator and its excellent physical decay characteristics (Cutler et al. 2013; Roesch and Riss 2010; Rösch 2013; Price and Orvig 2014). In the clinical routine, ^68^Ga-radiopharmaceuticals are used for the diagnosis of e.g. prostate cancer (^68^Ga-PSMA-11) or neuroendocrine tumors (^68^Ga-DOTA-TOC) using PET (Positron Emission Tomography) (Nelson et al. 2022). Besides ^68^Ga, other radioisotopes play a significant role in nuclear medicine. For example, ^177^Lu (t_1/2_ = 6.73 days, E_β-_ = 498 keV (78.6%), E_γ_ = 210 keV (11%) and 113 keV (6.4%) can be incorporated into ^177^Lu-radiopharmaceuticals like [^177^Lu]Lu-DOTA-TATE (Lutathera®) and [^177^Lu]Lu-PSMA-617 (Pluvicto®), which have reached approval to be used in clinics for therapeutic purposes in 2017 and 2022, respectively (Nelson et al. 2022). Additionally, ^198^Au is an exotic beta emitter (t_1/2_ = 2.7 days E_β- max_ = 0.96 MeV E_γ_ = 412 keV) and has potential for therapeutic applications due to its beta emission (Lever et al. 2003). To our knowledge, there are only a few reports on complexations reactions of ^198^Au, that used ^198^Au in the oxidation state of + 3 (Barnholtz et al. 2001; Maia et al. 2014). To date, only one complex with ^198^Au in the oxidation state + 1 has been reported (Kriel et al. 2015).

**Fig. 1:**
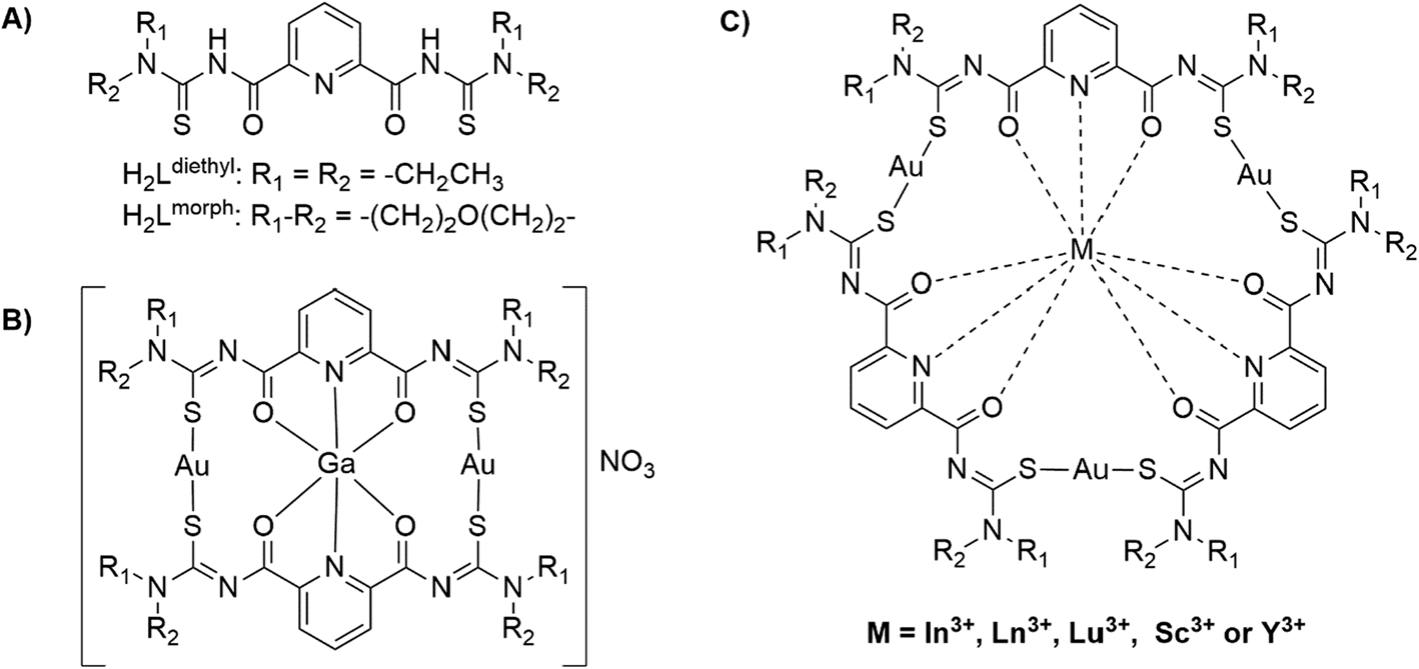
**A** Structure of the 2,6-dipicolinoylbis(*N,N*-dialkylthiourea) ligands used in this work, **B, C** gold(I) metallacages centered with M^3+^ ions

Aroyl-*N,N*-dialkylthioureas have affinity for metals in different oxidation states, besides having known pharmacological and biological properties (Maia et al. 2013; Salsi et al. 2019). The addition of a second bidentate *N*-acylthiourea linked symmetrically via a central pyridine gives rise to the so called 2,6-dipicolinoylbis(*N,N*-dialkylthioureas) (Fig. 1) and expands the possibilities for the coordination of metal ions and allows the formation of oligonuclear heterometallic complexes (Salsi et al. 2019; Nguyen et al. 2016; Le et al. 2019; Pham et al. 2019a, 2019b, 2017, 2020; Jesudas et al. 2020; Sucena et al. 2020). Due to the possibility to incorporate different metal ions, such novel systems are of interest for an application in nuclear medicine. Surprisingly, to our knowledge, only one work regarding the cytotoxicity assessment of zinc cages containing alkaline earth metal ions has been performed with such ligand systems which did not show relevant antiproliferative effects,(Le et al. 2019) even so no radiolabeling studies have been accomplished so far.

In a previous work, we described the chemistry of gold(I) metallacages derived from 2,6-dipicolinoylbis(*N,N*-ditheylthiourea) (H_2_L^diethyl^) and their guest M^3+^ metal (Sucena et al. 2023; Sucena 2018). Herein, we extend the library of metallacages by changing the peripheral moiety of the ligand by the morpholine motif (H_2_L^morph^). Additionally, the cytotoxicity of this new set of compounds was evaluated in comparison to the metallacages based on the diethyl motif ligand H_2_L^diethyl^. Finally, radiolabeling experiments from the uncoordinated H_2_L^diethyl^ and H_2_L^morph^ ligands as well as from their non-radioactive metallacages with ^68^Ga, ^177^Lu and ^198^Au(I) have been performed in an attempt to provide a theranostic platform, that can coordinate to a therapeutic and diagnostic radionuclide at the same time. The chelators standardly used for nuclear medicine application (e.g. DOTA), can only coordinate to one radionuclide. As such, two different molecules (e.g. [^177^Lu]Lu-DOTA-TOC and [^68^Ga]Ga-DOTA-TOC) are used in clinical application as a “theranostic”, although their biodistribution might differ.

## Results

### M^3+^ Complexes with 2,6-dipicolinoylbis(***N,N***-morpholinylthiourea) (H_2_L^morph^)

The oligonuclear coordination compounds are obtained from simple one-pot reactions of the ligands and mixtures of two metal ions with different Pearson's acidity, since the “soft” metal ion (Au^+^) will bind preferably to the sulfur atom, while the harder ions (M^3+^) will be directed to the center of the cage formed. In the present work, we used the H_2_L^morph^ ligand instead of H_2_L^diethyl^ which leads to the formation of neutral compounds of the composition [M{Au(L^morph^-κS)}_3_] (M = Y^3+^, Lu^3+^, Tb^3+^ and La^3+^) or to the cationic complex [Ga{Au(L^morph^-κS)}_2_]NO_3_ (see Fig. 1) in yields in the range from 33 to 62%. The [M{Au(L^morph^-κS)}_3_] compounds are only sparingly soluble in CH_2_Cl_2_, chloroform or DMSO and insoluble in MeOH, while [Ga{Au(L^morph^-κS)}_2_]NO_3_ is soluble in a 1:1 mixture of MeOH and CH_2_Cl_2_ or in DMSO. The complexes were characterized by means of elemental analysis, IR, ^1^H NMR (with the exception of the paramagnetic Tb^3+^ complex) and ESI^+^ mass spectrometry.

**Fig. 2:**
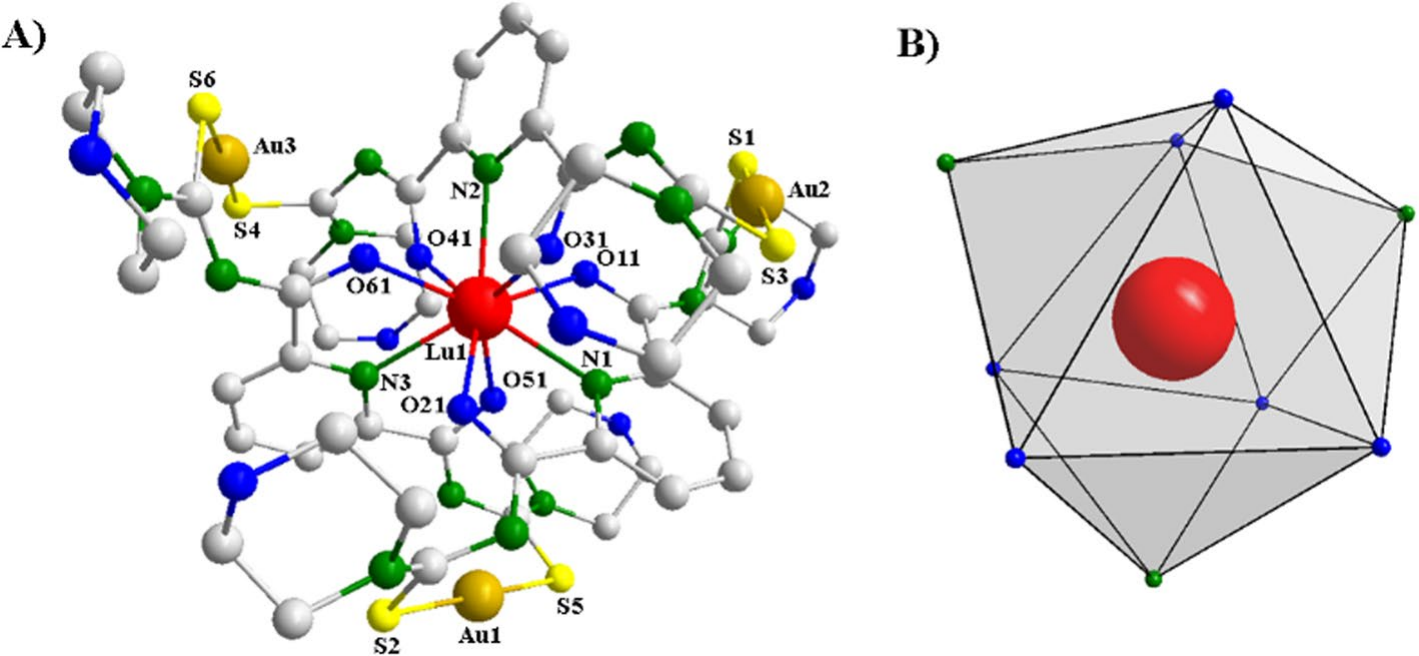
**A** Molecular structure of the complex [Lu{Au(L^morph^-κS)}_3_], as representative for the [M{Au(L^morph^-κS)}_3_]-type complexes. The hydrogen atoms and solvent molecules were omitted for clarity. **B** The coordination polyhedron around the Lu^3+^ metal center in [Lu{Au(L^morph^-κS)}_3_]. The nitrogen atoms are in green and oxygen atoms are in blue

The [M{Au(L^morph^-κS)}_3_] type complexes could be obtained in crystalline form after recrystallization from CH_2_Cl_2_/MeOH or CH_2_Cl_2_/MeCN and, therefore, had their crystal structures determined by single-crystal X-ray diffraction. Figure 2A shows the representation of the molecular structure of the [Lu{Au(L^morph^-κS)}_3_] complex as a representative of the {L^morph^}^2−^ containing compounds. The ORTEP representations of all compounds may be observed in the Supporting Information (Additional file 1: Figures S1.1–S1.4). Selected bond lengths and angles are shown in Additional file 1: Table S2. The complexes [M{Au(L^morph^-κS)}_3_] (M = Y, Tb and Lu) crystallize in the triclinic *P* $\overline{1 }$ space group, while [La{Au(L^morph^-κS)}_3_] crystallizes in the monoclinic space group* P*2_1_/*n*. All crystal structures contain co-crystallized solvent molecules of CH_2_Cl_2_, MeOH or MeCN. In all the complexes three bis(thiourea) ligands are doubly deprotonated and coordinate to the M^3+^ metal center as *NOO*-donors, leading to a coordination number of 9. The three ligand molecules are arranged in a helical fashion forming a coordination polyhedron around the lanthanide(III) ions which can be defined as a distorted tricapped trigonal prism (Fig. 2B) with the six oxygen donor atoms occupying the vertices of the prism and the three nitrogen atoms in equatorial plane.

### Cytotoxicity studies

Since gallium^3+^ and lutetium^3+^ play an important role in nuclear medicine as described in the introduction, the ^nat^gallium^3+^ and ^nat^lutetium^3+^ containing metallacages were selected for further assessment in in vitro cytotoxicity studies. The toxicity profile was evaluated on four different cell lines: MCF-7 (human breast cancer), PC-3 (prostate cancer) and the human glioblastoma cell lines U373 and U343. Table 1 shows the IC_50_ values [µM] for the selected metallacages and their uncoordinated ligands. Numerous gold compounds have been studied in the past towards their cytotoxic behavior with promising results (Casini and Messori 2011). As such, auranofin, an approved drug against arthritis and also known for anticancer activity, serves as a positive control (Marzo et al. 2017).

**Table 1:** IC_50_ values [µM] for the uncoordinated ligands, their gallium^3+^ and lutetium^3+^ metallacages and auranofin against four different tumor cell lines

Compounds	IC_50_ values (µM)			
	**MCF7**	**PC-3**	**U373**	**U343**
H_2_L^diethyl^	47.4 ± 4.0	34.3 ± 7.2	> 100	> 100
H_2_L^morph^	37.2 ± 4.4	41.8 ± 7.1	38.8 ± 2.0	43.2 ± 1.3
[Ga{Au(L^diethyl^)}_2_]NO_3_	4.6 ± 0.7	4.5 ± 0.7	6.8 ± 1.6	4.0 ± 1.1
[Ga{Au(L^morph^)}_2_]NO_3_	9.5 ± 2.3	11.1 ± 1.4	23.3 ± 6.5	22.9 ± 10.8
[Lu{Au(L^diethyl^)}_3_]	4.5 ± 0.2	2.3 ± 1.9	0.8 ± 0.6	1.2 ± 0.0
[Lu{Au(L^morph^)}_3_]	11.8 ± 1.7	6.7 ± 3.3	8.5 ± 3.1	5.0 ± 0.2
Auranofin	3.4 ± 1.9	5.0 ± 0.8	1.8 ± 0.9	1.1 ± 0.1

### Radiolabeling with ^68^Ga

**Fig. 3:**
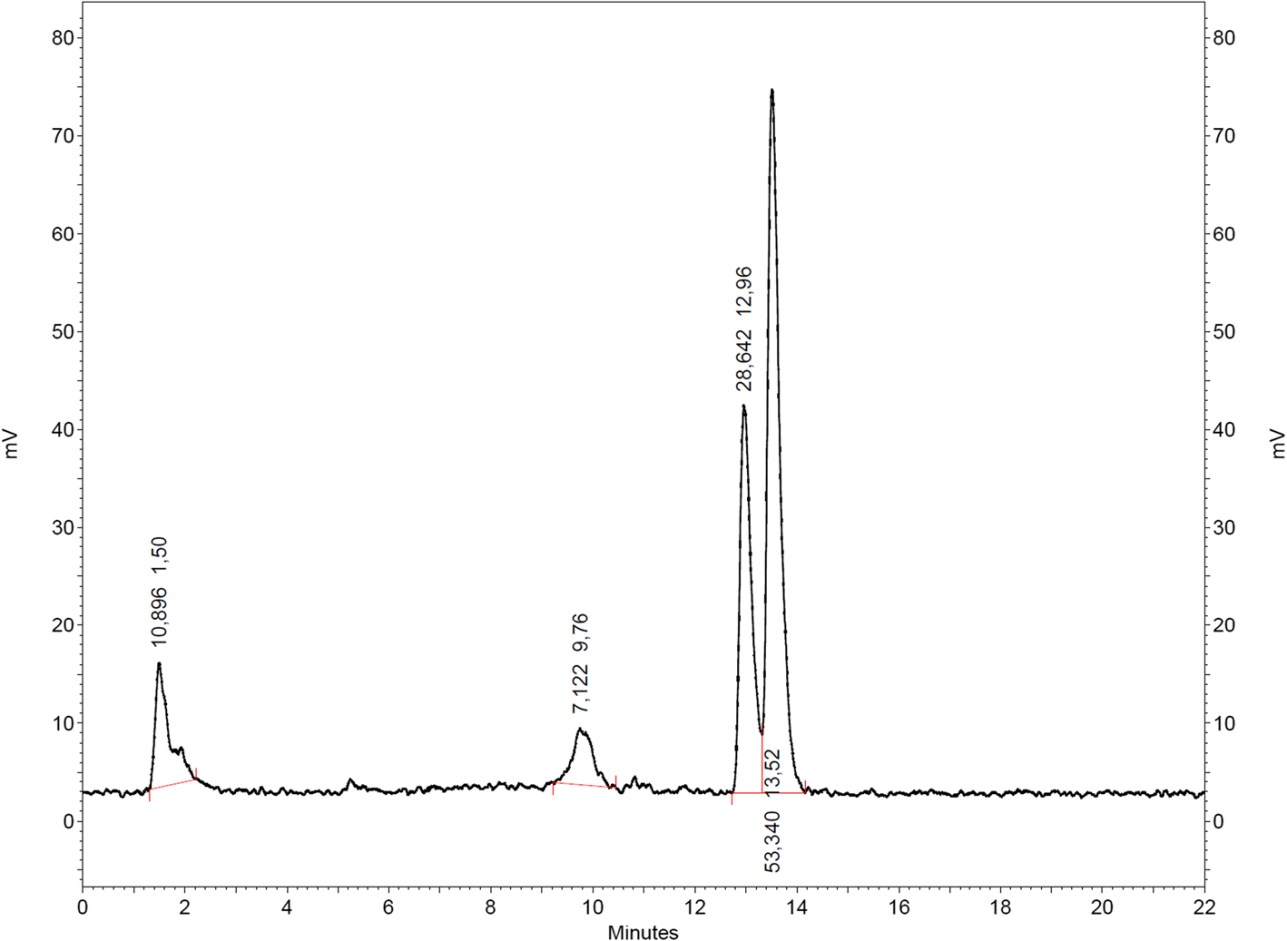
Radio-chromatogram of the reaction between H_2_L^diethyl^, [AuCl(THT)] and ^68^Ga in methanol/Na-acetate after 10 min of incubation time

The radiolabeling of H_2_L^diethyl^ or H_2_L^morph^ with ^68^Ga was performed by adding [^68^Ga]GaCl_3_ to a mixture of H_2_L^diethyl^ or H_2_L^morph^ and [AuCl(THT)] in methanol/Na-acetate buffer and incubating the reaction mixture for 10 min at room temperature. The complex [^68^Ga][Ga{Au(L^diethyl^)}_2_]^+^ at t_R_ = 13.0–13.5 min was received in 82% radiochemical purity (Fig. 3) as measured by radio-HPLC. The retention time of [^68^Ga][Ga{Au(L^diethyl^)}_2_]^+^ fits to the observed retention time of the non-radioactive standard (Additional file 1: Figure S3.1c). The first peak at t_R_ = 1.5 min can be attributed to free ^68^Ga and the peak at t_R_ = 9.8 min is most likely an intermediate as discussed in the Supporting Information (Page S20 and Additional file 1: Figure S3.2).

The radiolabeling of the non-radioactive complex [Ga{Au(L^diethyl^)}_2_]^+^ with ^68^Ga was also performed (Additional file 1: Figure S3.3b). Since the non-radioactive complex [Ga{Au(L^diethyl^)}_2_]^+^ is sparingly soluble in methanol, it was thus dissolved in DMSO and Na-acetate buffer was added for a stable pH of 4–5. In the radio-chromatogram, two very close peaks at t_R_ = 15.4 min and at t_R_ = 16.0 min were detected, representing the complex [^68^Ga][Ga{Au(L^diethyl^)}_2_]^+^.

**Fig. 4:**
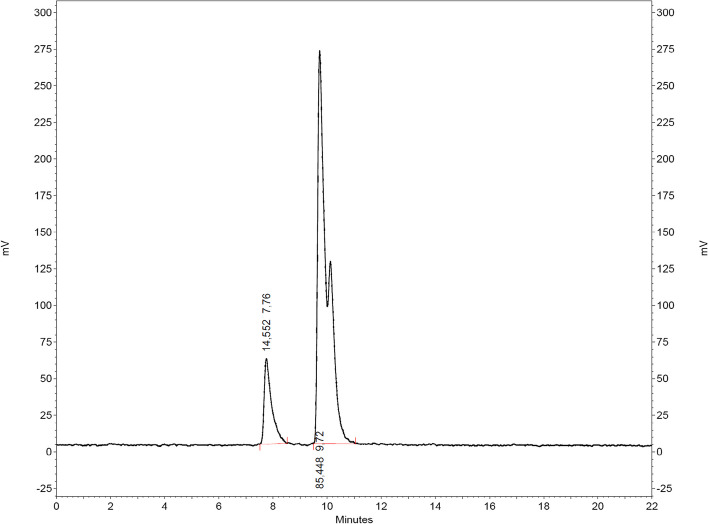
Radio-chromatogram of the reaction between H_2_L^morph^, [AuCl(THT)] and ^68^Ga in methanol and sodium acetate after 10 min

The complex [^68^Ga][Ga{Au(L^morph^)}_2_]^+^ was received in 86% radiochemical purity (Fig. 4) and a retention time of t_R_ = 9.7 min. At t_R_ = 7.8 min (15%), we observed an additional peak, that refers most likely to a radiolabeled intermediate. The observed retention times are in accordance with the non-radioactive standards (Additional file 1: Figure S3.4).

### Stability

**Fig. 5:**
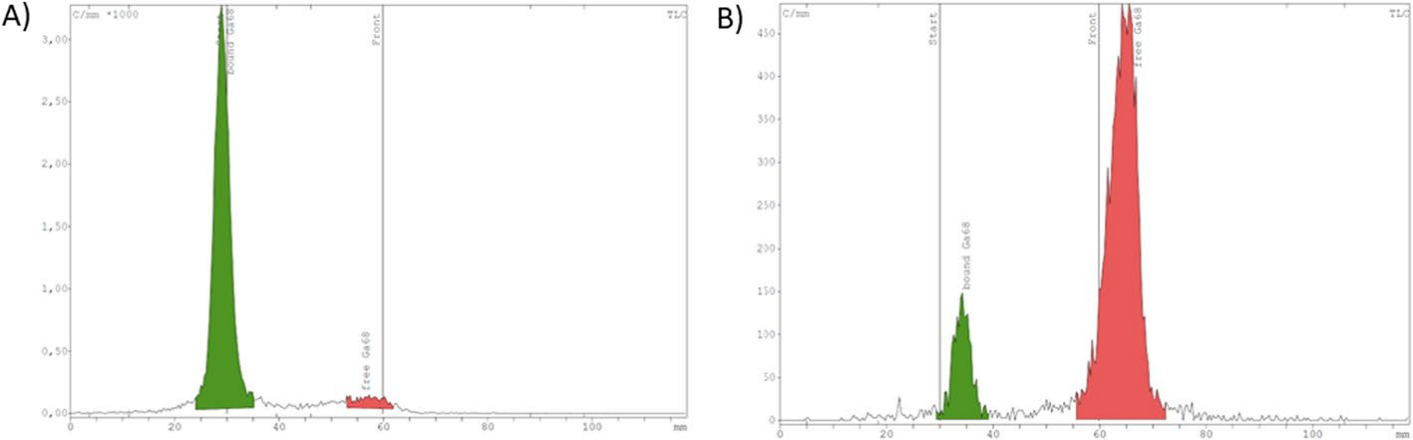
iTLC-chromatograms of stability assessment **A** [^68^Ga][Ga{Au(L^diethyl^)}_2_)]^+^ alone **B** [^68^Ga][Ga{Au(L^diethyl^)}_2_)]^+^ with human serum albumin

In order to assess the stability of [^68^Ga][Ga{Au(L^diethyl^)}_2_)]^+^ and [^68^Ga][Ga{Au(L^morph^)}_2_]^+^, the products were incubated for 30 min at room temperature in a 1:1 ratio with human serum albumin. As seen in Fig. 5 and Additional file 1: Figure S3.5, both complexes are not stable under the tested conditions, since the formation of free ^68^Ga (approx. 80%) appearing at the solvent front in the iTLC-chromatogram with citrate buffer as mobile phase was verified.

### Radiolabeling with ^198^Au

**Fig. 6:**
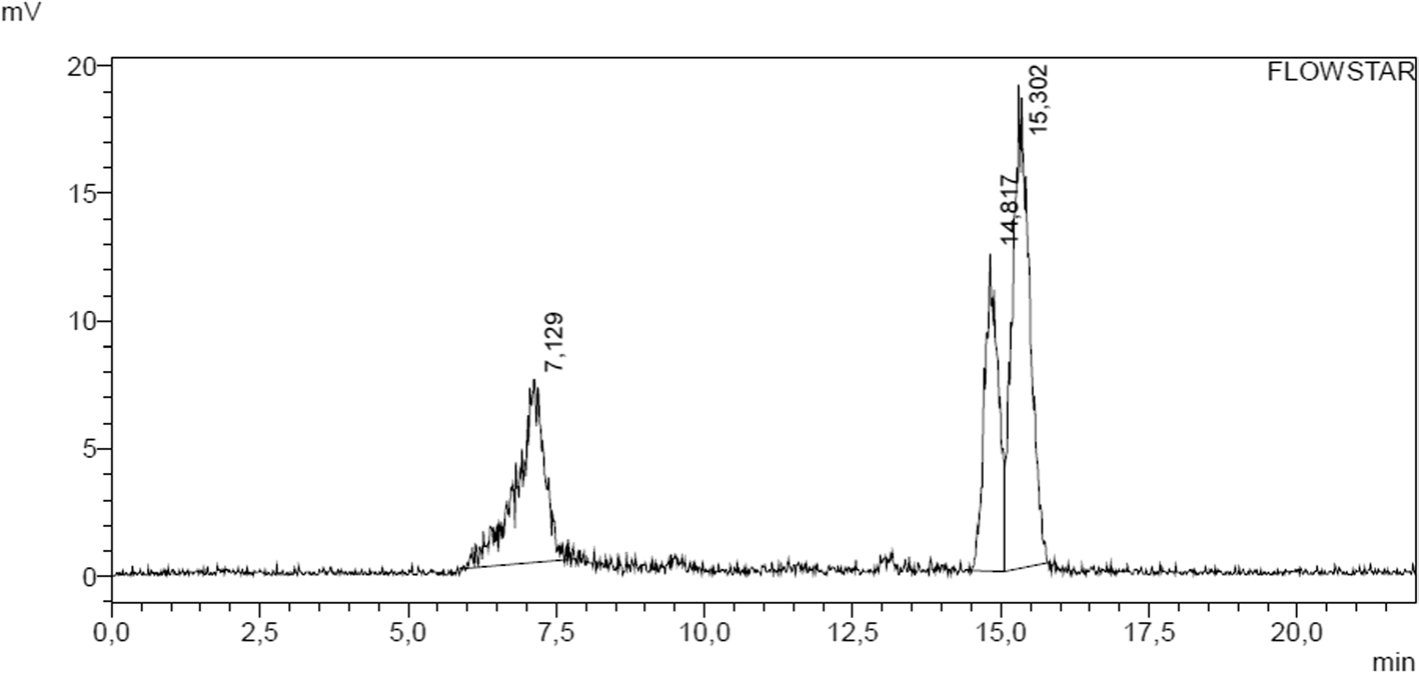
Radio-chromatogram of [^198^Au][Ga{Au(L^diethyl^)}_2_]^+^

[^198^Au][Ga{Au(L^diethyl^)}_2_]^+^ and [^198^Au][Ga{Au(L^morph^)}_2_]^+^ were synthesized following the same protocol as described in the Experimental section. After adding [^198^Au]AuCl(THT)] to a solution of the isolated complex e.g. [Ga{Au(L^diethyl^)}_2_]NO_3_ in DMF, the solution was incubated for 10 min at 90 °C. In the radio-HPLC chromatogram of [^198^Au][Ga{Au(L^diethyl^)}_2_]^+^ (Fig. 6), peaks at t_R_ = 7.1 min (29%, [^198^Au]AuCl(THT)]) and t_R_ = 14.8–15.3 (71%, complex) were observed. The complex [^198^Au][Ga{Au(L^diethyl^)}_2_]^+^ was received in a total of 71.2% radiochemical purity and a specific molar activity of 150 MBq/nmol. A chromatogram of the starting material [^198^Au]AuCl(THT)] can be found in Additional file 1: Figure S3.6.

In the radio-HPLC chromatogram of [^198^Au][Ga{Au(L^morph^)}_2_]^+^ (Additional file 1: Figure S3.7), we observe a peak at t_R_ = 7.1 (67%, [^198^Au]AuCl(THT)), t_R_ = 9.5 min (2.1%, intermediate), t_R_ = 10.3 min (3%, intermediate) and t_R_ = 11.5–12.0 (28%, complex). The complex [^198^Au][Ga{Au(L^morph^)}_2_]^+^ was received in a total of 28% radiochemical purity and a specific molar activity of 157 MBq/nmol.

### Radiolabeling with ^177^Lu

**Fig. 7:**
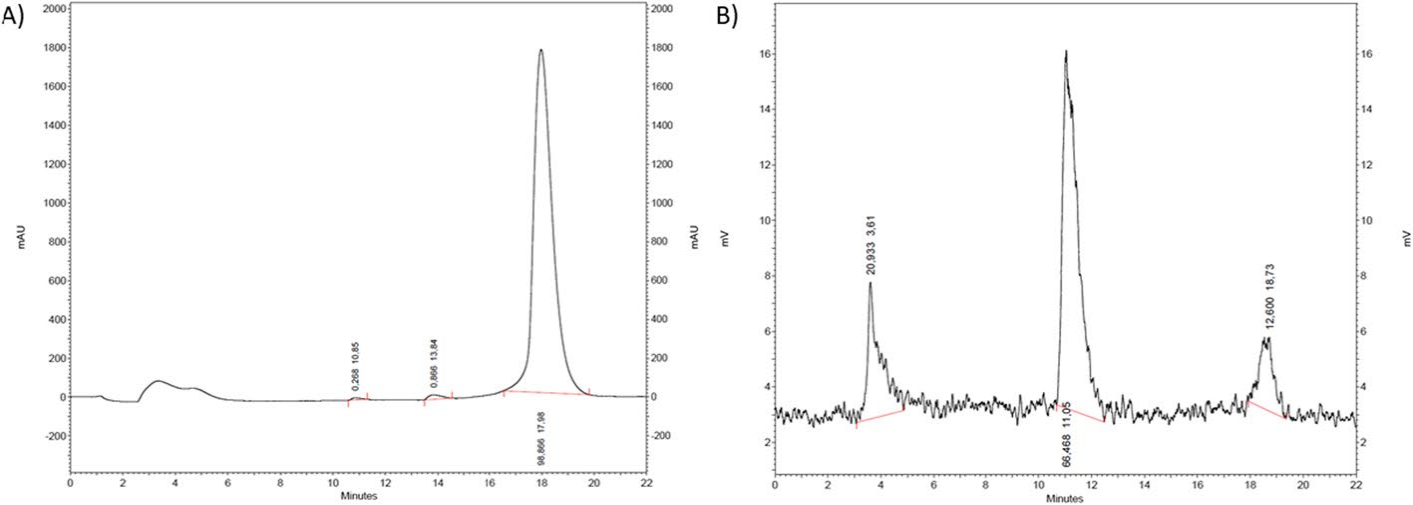
**A** UV-chromatogram of [^nat^Lu{Au(L^diethyl^)}_3_], **B** radio-HPLC chromatogram [^177^Lu][Lu{Au(L^diethyl^)}_3_]

[^177^Lu][Lu{Au(L^diethyl^)}_3_] and [^177^Lu][Lu{Au(L^morph^)}_3_] were synthesized by adding [^177^Lu]LuCl_3_ to a solution of the respective ligand H_2_L^diethyl^ or H_2_L^morph^ and [AuCl(THT)] in DMF and ascorbate buffer. After incubation for 10 min at room temperature, we analyzed the reactions mixtures. The radio-chromatogram of [^177^Lu][Lu{Au(L^diethyl^)}_3_] shows a peak at t_R_ = 3.6 min (free ^177^Lu), one at t_R_ = 11.0 min (intermediate) and one at t_R_ = 18.2 min ([^177^Lu][Lu{Au(L^diethyl^)}_3_], 17%) (Fig. 7B). The successful radiolabeling of the complex is supported by the non-radioactive standard in Fig. 7A. The radio-chromatogram of [^177^Lu][Lu{Au(L^morph^)}_3_] shows a peak at t_R_ = 1.9–3.8 min (free ^177^Lu) and one at t_R_ = 12.3 min ([^177^Lu][Lu{Au(L^morph^)}_3_], 24%) (Additional file 1: Figure S3.8).

## Discussion

The complexes [Lu ⊂ {Au_3_(L1^ethyl^)_3_}] and [Ga ⊂ {Au_2_(L1^ethyl^)_2_}]NO_3_ were prepared by one-pot reactions of each three and two equivalents, respectively, of [AuCl(tht)] (tht = tetrahydrothiophene) and H_2_L1^ethyl^, with one equivalent of the nitrates of the trivalent metal ions. Details about the syntheses of these complexes have been previously discussed (Sucena et al. 2023; Sucena 2018).

The relatively lower yields obtained for H_2_L^morph^, specifically for the lutetium derivative, compared to the H_2_L^diethyl^ reactions(Sucena et al. 2023) is related to the formation of insoluble polymeric products which seems to be a consequence of the additional oxygen donor atom in the morpholine moiety (Nguyen et al. 2016). Fortunately, these by-products could be easily removed by recrystallization methods. A discussion about the spectroscopic and spectrometric characterization (Additional file 1: Figures S2.1–S2.12) has been made previously with H_2_L^diethyl^ derivatives and shall not be a matter of the present work.

Regarding the geometries of the synthesized complexes, the soft sulfur donor atoms of the ligands are bond to three gold centers in an almost linear geometry with the S–Au-S angle in the range between 175.04 and 179.56°. The Au–S bond lengths present values between 2.275 and 2.293 Å without a considerable influence of the M^3+^ metal center. On the other hand, the M^3+^–O and M^3+^–N bond lengths are clearly shorter for the Lu^3+^ complex which also leads to modifications on the bond angles (Additional file 1: Table S2). This is easily explained by the different ionic radii due to the lanthanide contraction effects (Bart 2023). A significant increase in the C=O and C=S bond lengths as well as a shortening of the CN bonds is observed upon coordination to the metal ions, which is in accord to the FT-IR spectra.

Regarding the cytotoxicity studies, the uncoordinated ligands H_2_L^diethyl^ an H_2_L^morph^ do not have toxic effects on the tested cancer cell lines. To note, the metallacages [Ga{Au(L^diethyl^)}_2_]NO_3_ and [Lu{Au(L^diethyl^)}_3_] show a similar toxicity to auranofin and an approximate tenfold increased toxicity compared to their uncoordinated ligands. On the contrary, the metallacage [Ga{Au(L^morph^)}_2_]NO_3_ is less toxic compared to [Ga{Au(L^diethyl^)}_2_]NO_3_, while [Lu{Au(L^morph^)}_3_] is approx. twofold less toxic compared to [Lu{Au(L^diethyl^)}_3_]. Although a morpholine moiety is often used to increase the cytotoxicity of the respective compound,(Kumari and Singh 2020) we observe the opposite effect for our tested metallacages. It could be hypothesized, that the uptake of the metallacages with the morpholine moiety is altered or the target interaction is disturbed. To confirm this, further studies are needed in order to investigate the mechanisms of action for our compounds. Previously reported [MZn_2_(μ-AcO)_2_(L^diethyl^)_2_] and [MZn_2_(L^diethyl^)_3_] (M = Ca or Ba) presented IC_50_ values in the order of 35 µM or higher,(Le et al. 2019) which indicates that the metal ions are responsible for the cytotoxic effects in the present work.

Regarding the radiolabeling studies with ^68^Ga, the presence of two peaks with [^68^Ga][Ga{Au(L^diethyl^)}_2_]^+^ may be explained by the presence of two different conformations of the complex. The formation of isomers in different conformations is due to the rigidity of the formed assemblies and hindered rotation around the C-NEt_2_ bonds. This fact has been observed in many other complexes with {L1^ethyl^}^2−^,(Nguyen et al. 2016; Jesudas et al. 2020; Pham et al. 2017, 2020; Sucena et al. 2020, 2023) including chelate complexes with the parent benzoylthioureato ligands, where a rotational barrier of approximately 650 kJ/mol has been found (Kleinpeter and Beyer 1975). This is in accord with the ^1^H NMR of the {L1^ethyl^}^2−^ derivatives which do not show equivalent the protons of the methylene groups and also with the possibility of different conformers for morpholine derivatives (Xie et al. 2011).

Interestingly, when performing the experiment in DMSO/Na-acetate at pH 4–5 instead of methanol/Na-acetate, we observed an increased formation of the intermediate [^68^Ga][GaAu(L^diethyl^)_2_] (31%, Additional file 1: Figure S3.3) and the complex [^68^Ga][Ga{Au(L^diethyl^)}_2_]^+^ (65%). DMSO is a nucleophilic, aprotic solvent, whereas methanol is a protic solvent. The formation of the complex [^68^Ga][Ga{Au(L^diethyl^)}_2_]^+^may take place preferably in protic solvents with a radiochemical purity of 82% in comparison to the aprotic solvent with a radiochemical purity of 65%.

A good match between the HPLC chromatograms of the cold and radioactive analogues was found only for the pair [^nat^Ga][Ga{Au(L^diethyl^)}_2_]^+^ and [^68^Ga][Ga{Au(L^diethyl^)}_2_]^+^, and for the pair [^nat^Ga][Ga{Au(L^morph^)}_2_]^+^ and [^68^Ga][Ga{Au(L^morph^)}_2_]^+^, thus indicating that, presumably, the structures of the two analogous compounds are identical. However, it should be noted that the conventional procedure usually followed to obtain the strongest evidence for this conclusion involves co-injection of two compounds into the same HPLC column equipped with an in-line array of radioactive and UV detectors.

## Conclusions

In this work, we successfully synthesized and fully characterized a new set of metallacages of the composition [M{Au(L^morph^-κS)}_3_] (M = La^3+^, Tb^3+^, Lu^3+^ or Y^3+^) and [Ga{Au(L^morph^-κS)}_2_]NO_3_. Due to their ability to incorporate different metals that are interesting for nuclear medicine application, their antiproliferative effects in four human cancer cell lines and their radiolabeling behavior were also evaluated.

Regarding their cytotoxic profile, the IC_50_ [µM] values of [Ga{Au(L^diethyl^)}_2_]NO_3_, [Ga{Au(L^morph^)}_2_]NO_3_, [Lu{Au(L^diethyl^)}_3_] and [Lu{Au(L^morph^)}_3_] were determined. The four compounds show similar values compared to their gold standard auranofin. To note, the metallacages derived from H_2_L^diethyl^ are more cytotoxic than their counterparts with the H_2_L^morph^ ligands. Further studies are needed to elucidate their mechanism of action.

In terms of radiolabeling experiments, the ^68^Ga radiolabeling lead to high radiochemical purities of [^68^Ga][Ga{Au(L^diethyl^)}_2_]^+^ and [^68^Ga][Ga{Au(L^morph^)}_2_]^+^. On the other hand, the ^177^Lu radiolabeling afforded the complexes [^177^Lu][Lu{Au(L^diethyl^)}_3_] and [^177^Lu][Lu{Au(L^morph^)}_3_], but in low radiochemical purities. Starting from [^198^Au]AuCl(THT)], the species [^198^Au][Ga{Au(L^diethyl^)}_2_]^+^ and [^198^Au][Ga{Au(L^morph^)}_2_]^+^ were successfully prepared. These metallacages are not stable in human serum. Most likely, the Au(I) gets bound by the albumins and thus the metallacage gets disrupted. Presently, studies with further variations of metal ions are underway in our laboratories in order to obtain more stable complexes in human serum. Overall, we showed that different metal radionuclides (^68^Ga, ^177^Lu and ^198^Au) can be inserted in the system. Another interesting aspect that will be evaluated in future studies is the complexation of ^198^Au in the oxidation state +3, as such complexes have also been studied towards their cytotoxic behavior in biological systems (Casini and Messori 2011).

## Methods

### Materials

All chemicals were reagent grade and used without further purification unless otherwise stated. [AuCl(THT)] was synthesized according to a standard procedure from HAuCl_4_ and tetrahydrothiophene in ethanol (Uson et al. 1989). THF was distilled over sodium, acetone was distilled over MgSO_4_ and Et_3_N over NaOH. The reactions within the context of ligand synthesis with moisture-sensitive compounds were performed under an argon atmosphere using standard Schlenk techniques. The ligands were prepared as previously reported (Yokoyama et al. 1990; Schröder et al. 2000; Rodenstein et al. 2008).

Solvents for HPLC were obtained as HPLC grade. TraceSelect water (Sigma-Aldrich) was used in radiolabeling experiments. The pharmaceutical grade ^68^Ge/^68^Ga generator (GalliaPharm®, Eckert & Ziegler Radiopharma GmbH, Germany), and Lutetium-177 n.c.a. (EndolucinBeta, ITM, Garching, Germany) were used as radiochemical precursors. ^198^Au(0) was prepared at the research reactor TRIGA Mainz, Germany. Activity counting was performed using a borehole counter (Nuklear-Medizintechnik Dresden GmbH, Germany). HPLC (^68^Ga or ^177^Lu) was performed using the HPLC system Knauer Azura (UVD: 2.1 L; P6.1L) coupled with UV and radiometric (Raytest Socket 2″8103 0370) detectors. HPLC (^198^Au) was performed using a Shimadzu gradient system (Kyoto, Japan) equipped with a SPD-20A UV/Vis detector. Radioactivity was detected via a HERM LB 500 NaI detector and a Flowstar2 LB514 detector (Berthold Technologies, Bad Wildbad, Germany). The TLC scanner MiniGita from Raytest was used. The column for ^68^Ga, ^177^Lu and ^198^Au experiments used for radio-HPLC (RSC Gel C18ec, 125 × 4.0 mm, 5 μm) was purchased from R. Sauerbrey Chromatographie (D-Reinhardshagen). Eluents for all HPLC operations were water (solvent A) and acetonitrile (solvent B), both containing 0.1 vol.% trifluoroacetic acid (TFA). The gradient used was 0–15 min 0–100% B, 15–20 min 100% B, 20–22 min 100–0% B. The purity of the compounds tested in vitro was performed via HPLC and was > 95%.

### Physical measurements

The IR spectra were recorded on a Thermo Scientific Nicolet iS10 FTIR spectrometer in the range of 4000–400 cm^−1^. The ^1^H NMR of the compounds were measured on a JEOL 400 MHz spectrometer. Elemental analyses of carbon, hydrogen, nitrogen and sulfur were determined using a Heraeus vario EL elemental analyzer. The mass spectra were measured with an Agilent 6210 ESI-TOF spectrometer (Agilent Technologies, Santa Clara, CA, USA). The flow rate was 4 μL/min and the spray voltage was 3.8 kV and the desolvation gas was set at 15 psi. Some representative spectra of the IR, NMR and MS spectra are given as Supporting Information.

### X-ray crystallography

The intensities for the X-ray determinations for all the other complexes were collected at 200 K on a STOE IPDS 2T instrument with Mo-Kα radiation (λ = 0.71073 Å) using a graphite monochromator and applying X-RED32 for the absorption corrections (X-RED32, STOE Cie GmbH 2002). The structure solutions were performed with the SHELXS 97 (Sheldrick 2008) or SHELXT (Sheldrick 2015) and refined with SHELXL 2016/4 (Sheldrick 2015) programs included in the Olex 2 program package (Dolomanov et al. 2009). The representation of molecular structure of the complex 1 was done using the program DIAMOND 4(Brandenburg 2018) and the ellipsoid plots were prepared with Mercury 4.3.1 (Macrae et al. 2020). More details on data collections and structure calculations are given in Additional file 1: Table S1.

### Synthesis of the complexes

#### [M{Au(L^morph^-*κS*)}_3_]

To a suspension of [AuCl(THT)] (48*.*1 mg, 0*.*15 mmol) and M(NO_3_)_3_*·*nH_2_O (M = La, Tb or Lu) or Y(CF_3_SO_3_)_3_ (0*.*05 mmol) in MeOH (3 mL) was added H_2_L^morph^ (63.5 mg, 0.15 mmol). After stirring the reaction mixture for 30 min at room temperature, 6 drops of Et_3_N were added which led to the formation of colorless or yellow precipitates. After stirring the reaction mixtures for additional 2 h, the precipitates were collected, washed with *n*-hexane and recrystallized from CH_2_Cl_2_/MeOH (3:1). The obtained crystalline solids were filtered, washed with little *n*-hexane and dried under reduced pressure.

#### [La{Au(L^morph^-*κS*)}_3_] (1)

Yield: 46.0 mg (46%). Elemental Analysis calcd for C_51_H_57_Au_3_LaN_15_O_12_S_6_∙CH_2_Cl_2_: C, 30.0; H, 2.9; N, 10.1; S, 9.3%. Found: C, 29.4; H, 3.0; N, 10.4; S, 9.4%.

IR (ATR, cm^−1^): 2966 (w), 2918 (w), 2851 (w), 1578 (m), 1549 (vs), 1503 (br), 1436 (vs), 1389 (s), 1288 (s), 12 224 (s), 1105 (s), 1024 (s), 935 (m), 749 (m), 664 (m), 632 (m).

^1^H NMR (400 MHz, CDCl_3_, ppm): δ 8.16 (d, J = 7.7 Hz, 6H, Py), 7.94 (t, J = 7.7 Hz, 3H, Py), 4.00–3.60 (m, 48H, CH_2_).

ESI^+^ MS (*m/z*): 1994.0813, [M + H]^+^ (calcd. 1994.0729); 2016.0628, [M + Na]^+^ (calcd. 2016.0588); 2032.0375, [M + K]^+^, (calcd. 2032.0332).

#### [Tb{Au(L^morph^-*κS*)}_3_] (2)

Yield: 62.0 mg (62%). Elemental Analysis Calcd for C_51_H_57_Au_3_N_15_O_12_S_6_Tb: C, 30.4; H, 2.8; N, 10.4; S, 9.5%. Found: C, 29.9; H, 2.9; N, 10.6; S, 9.5%.

IR (ATR, cm^−1^): 3447 (vw), 2963 (w), 2914 (w), 2849 (w), 1583 (m), 1553 (vs), 1510 (vs), 1436 (s), 1394 (s), 1286 (s), 1105 (s), 1023 (s), 941 (m), 743 (m), 663 (m), 631 (m).

ESI^+^ MS (m/z): 2014.092, [M + H]^+^, (calcd. 2014.096); 2036.068, [M + Na]^+^, (calcd. 2036.078); 2052.044, [M + K]^+^, (calcd. 2052.052).

#### [Lu{Au(L^morph^-*κS*)}_3_] (3)

Yield: 33.0 mg (33%). Elemental Analysis Calcd for C_51_H_57_Au_3_LuN_15_O_12_S_6_∙0.5CH_2_Cl_2_: C, 29.8; H, 2.8; N, 10.2; S, 9.3%. Found: C, 29.5; H, 2.9; N, 10.4; S, 9.4%.

IR (ATR, cm^−1^): 2966 (w), 2913 (w), 2847 (w), 1586 (m), 1557 (vs), 1512 (vs), 1436 (s), 1396 (s), 1287 (s), 1224 (s), 1104 (s), 1020 (m), 942 (m), 742 (m), 661 (m), 631 (m).

^1^H NMR (400 MHz, CDCl_3_, ppm): δ 8.16 (m, 6H, Py), 7.97 (t, J = 7.7 Hz, 3H, Py), 3.82–3.60 (m, 48H, CH_2_).

ESI^+^ MS (m/z): 2030.106, [M + H]^+^, (calcd. 2030.112); 2052.068, [M + Na]^+^, (calcd. 2052.094); 2093.006, [M + Na + MeCN]^+^, (calcd. 2093.120).

#### [Y{Au(L^morph^-*κS*)}_3_] (4)

Yield: 50.0 mg (51%). Elemental Analysis Calcd for C_51_H_57_Au_3_N_15_O_12_S_6_Y∙0.5CH_2_Cl_2_: C, 31.1; H, 2.9; N, 10.6; S, 9.7%. Found: C, 30.5; H, 3.0; N, 10.9; S, 9.8%.

IR (ATR, cm^−1^): 2966 (w), 2914 (w), 2849 (w), 1584 (m), 1556 (vs), 1511 (vs), 1436 (s), 1401 (s), 1286 (s), 1104 (s), 1019 (s), 941 (m), 834 (m), 743 (m), 662 (m), 630 (m).

^1^H NMR (400 MHz, CDCl_3_, ppm): δ 8.15 (d, J = 7.7 Hz, 6H, Py), 7.96 (t, J = 7.7 Hz, 3H, Py), 3.82–3.60 (m, 48H, CH_2_).

ESI^+^ MS (m/z): 1944.229, [M + H]^+^, (calcd. 1944.077); 2007.970, [M + Na + MeCN]^+^, (calcd. 2007.085).

#### [Ga{Au(L^morph^-κS)}_2_](NO_3_) (5)

The ligand H_2_L^morph^ (42.4 mg, 0.10 mmol) was added to a suspension of [AuCl(THT)] (32.1 mg, 0.10 mmol) and Ga(NO_3_)_3_·xH_2_O (12.8 mg, 0.05 mmol) in MeOH (3 mL). Stirring of the reaction mixtures for 30 min at room temperature was followed by the addition of 3 drops of Et_3_N. This led to the formation of a yellow precipitate which was filtered, washed with *n*-hexane and dried under vacuum.

Yield: 38.5 mg (56%).

IR (ATR, cm^−1^): 3461 (w), 3080 (vw), 2961 (w), 2905 (w), 2851 (w), 1609 (s), 1579 (s), 1505 (br), 1433 (m), 1385 (s), 1288 (br), 1261 (s), 1110 (s), 1025 (s), 947 (m), 760 (m), 674 (m), 632 (m).

ESI^+^ MS (m/z): 1305.0373, [M]^+^, (calcd. 1305.0338).

### Cell viability studies

The human pancreatic cancer cell line PC-3, the human breast cancer cell line MCF7 and the human glioblastoma cell lines U373 and U343 were kindly provided by BERIC. Cells were cultured in RPMI 1640 containing GlutaMax, supplemented with 10% FBS and 1% penicillin/streptomycin (all from Invitrogen), at 37 °C under a humidified atmosphere of 95% of air and 5% CO_2_ (Heraeus, Germany).

For evaluation of growth inhibition, cells were seeded in 96-well plates (Costar, Integra Biosciences, Cambridge, MA) at a concentration of 10,000 cells per well (PC-3, U373 and U343) or 8000 cells per well (MCF7) and grown for 24 h in complete medium. Solutions of the gold(I) cages were prepared by diluting a freshly prepared stock solution (10^−2^ M in DMSO, DMF for [Lu{Au(L^morph^)}_3_]) of the corresponding compound in aqueous media (RPMI). Auranofin was purchased from Sigma-Aldrich and stock solutions were prepared in water. Afterwards, the intermediate dilutions of the compounds in the cell culture medium were added to the wells (200 μL) to obtain a final concentration ranging from 0 to 100 μM, and the cells were incubated for 72 h. Afterwards, 3-(4,5-dimethylthiazol-2-yl)-2,5-diphenyltetrazoliumbromide (MTT) was added to the cells at a final concentration of 0.5 mg ml^−1^ and incubated for 2 h, then the culture medium was removed and the violet formazan (artificial chromogenic precipitate of the reduction of tetrazolium salts by dehydrogenases and reductases) dissolved in DMSO. The optical density of each well (96-well plates) was quantified in quadruplicates at 550 nm using a multi-well plate reader, and the percentage of surviving cells was calculated from the ratio of absorbance of treated to untreated cells. The IC_50_ value was calculated as the concentration reducing the proliferation of the cells by 50% and it is presented as a mean (± SE) of at least three independent experiments by using GraphPadPrism 8.

### Radiolabeling experiments

#### Radiolabeling with ^68^Ga

For the manual labeling of the ligands H_2_L^diethyl^ and H_2_L^morph^, a 2 mL glass reaction vial was used. 100 µL of H_2_L^diethyl^ or H_2_L^morph^ (1 mg /100 µL in methanol) were added to 500 µL Na-acetate buffer (1.85M) and mixed with 100 µL ^68^Ga eluate from an approved ^68^Ge/^68^Ga generator GalliaPharm® (Eckert&Ziegler) (≈ 140 MBq) and incubated for 10 min at room temperature (or at 90°C with DMSO as solvent).

#### Radiolabeling with ^177^Lu

For the manual labeling of the ligands H_2_L^diethyl^ and H_2_L^morph^, a 2 mL glass reaction vial was used. 100 µL of H_2_L^diethyl^ or H_2_L^morph^ (1 mg /100 µL in DMF) were added to 500 µL ascorbate buffer (0.1 M) and mixed with 100 µL n.c.a. ^177^LuCl_3_ (EndolucinBeta, ITM) (≈ 200 MBq) and incubated for 10 min at room temperature.

#### Radiolabeling with ^198^Au

### Preparation of [^198^Au]AuCl(THT)]

Solid gold bars of 1.4 mg and 2.8 mg weight were irradiated for 11 and 4 min, respectively in the fast rabbit system of the research reactor TRIGA Mainz at a thermal power of 100 kW. With a neutron flux of 1.6 × 10^12^ n/cm^2^ s^−1^ 1.3 MBq and 1 MBq of ^198^Au were produced with these bars.

The [^198^Au]Au(0) was dissolved in 40 µL aqua regia and heated up for 2 min at 90 °C until complete dissolution. Afterwards, the aqua regia was evaporated to dryness. The solid was redissolved in 400 µL ultra pure water and 200 µL ethanol to give a yellow solution of HAuCl_4_. 10 µL of tetrahydrothiophene was added and a colour change from yellow to colorless was observed. In total, we have 610 µL of [^198^Au]AuCl(THT)] that can be used without further purification.

### Radiolabeling of the metallacages

For the manual labeling of the metallacages with ^198^Au, a 2 mL glass reaction vial was used. 100 µL of the [Ga{(Au)Au(L^diethyl^)}_2_]^+^ and [Ga{(Au)Au(L^morph^)}_2_]^+^ (1mg/100 µL in DMF) was mixed with 100 µL [^198^Au]AuCl(THT)] (≈ 120 KBq) and heated up to 90 °C for 10 min.

### Stability of ^68^Ga-complexes

100 µL of product solution from either [^68^Ga][Ga{Au(L^diethyl^)}_2_]^+^ or [^68^Ga][Ga{Au(L^morph^)}_2_]^+^, was incubated at room temperature for 30 min with 100 µL of human serum albumin. The radiochemical purity was assessed via iTLC measurement as described in the quality control section.

### Quality control

#### ^68^Ga/^177^Lu

20 µL of product solution was manually injected into the HPLC system from Knauer.

For the ITLC, 1 µL of sample was spotted on silica gel plates and citrate buffer was used as mobile phase. After development of the plates, radioactivity distribution was assessed with a TLC scanner.

#### ^198^Au

40 µL of the final preparation was diluted with water to 90 µL which were injected into the HPLC system from Shimadzu.

## Supporting information

Crystal structures, refinement data, FTIR spectra, ^1^H NMR spectra and ESI MS spectra, molecular formula strings, HPLC UV-chromatograms and radio-chromatograms can be found in the Supporting Information. CCDC 2,258,607−2,258,610 contain the supplementary crystallographic data for this paper. These data can be obtained free of charge via www.ccdc.cam.ac.uk/data_request/cif, or by emailing data_request@ccdc.cam.ac.uk, or by contacting The Cambridge Crystallographic Data Centre, 12 Union Road, Cambridge CB2 1EZ, UK; fax: + 44 1223 336033.

### Supplementary Information

**Additional file 1**. Supplementary Information.

## Data Availability

The datasets used and/or analyzed during the current study are available from the corresponding author on reasonable request.

## Abbreviations

EA: Elemental analysis
ESI–MS: Electrospray ionization mass spectrometry
^1^H NMR: Proton nuclear magnetic resonance spectroscopy
IR: Infrared
iTLC: Instant thin layer chromatography
PSMA: Prostate specific membrane antigen
THT: Tetrahydrothiophene

## References

[CR1] Abram U, Alberto R (2006). Technetium and rhenium—coordination chemistry and nuclear medical applications. J Braz Chem Soc.

[CR2] Barnholtz SL, Lydon JD, Huang G, Venkatesh M, Barnes CL, Ketring AR, Jurisson SS (2001). Syntheses and characterization of gold(III) Tetradentate schiff base complexes. X-ray crystal structures of [Au(sal2pn)]Cl·25H2O and [Au(sal2en)]PF6. Inorg Chem.

[CR3] Bart SC (2023). What is the “lanthanide contraction”?. Inorg Chem.

[CR4] Brandenburg K. Diamond crystal and molecular structure visualization (version 4.0.2). Crystal Impact GbR, Bonn, Germany (2018).

[CR5] Casini A, Messori L (2011). Molecular mechanisms and proposed targets for selected anticancer gold compounds. Curr Top Med Chem.

[CR6] Cutler CS, Hennkens HM, Sisay N, Huclier-Markai S, Jurisson SS (2013). Radiometals for Combined imaging and therapy. Chem Rev.

[CR7] Dilworth JR, Pascu SI, Long N, Wong W-T (2015). The radiopharmaceutical chemistry of technetium and rhenium. The Chemistry of molecular imaging.

[CR8] Dolomanov O, Bourhis L, Gildea R, Howard J, Puschmann H (2009). OLEX2: a complete structure solution, refinement and analysis program. J Appl Cryst.

[CR9] Gielen M, Tiekink ERT (2005). Metallotherapeutic drugs and metal-based diagnostic agents: the use of metals in medicine.

[CR10] Jesudas JJ, Pham CT, Hagenbach A, Abram U, Nguyen HH (2020). Trinuclear CoIILnIIICoII complexes (Ln = La, Ce, Nd, Sm, Gd, Dy, Er, and Yb) with 2,6-dipicolinoylbis(N, N-diethylthiourea): synthesis, structures, and magnetism. Inorg Chem.

[CR11] Kleinpeter E, Beyer L (1975). ^1^H-NMR-Untersuchung der behinderten rotation um die C-N-Bindung in 1,1’-Diäthyl-3-benzoylharnstoff-Derivaten. J Prakt Chem.

[CR12] Kriel FH, Szucs Z, van Staden JA, Bester CJ, Mongane M, Lamprecht S, Rae WID, Zeevaart JR (2015). Biodistribution of a potential chemotherapeutic, dinuclearbisphosphinogold(I) dithiocarbamate, as determined by its ^198^Au radiolabelled analogue. J Radioanal Nucl Chem.

[CR13] Kumari A, Singh RK (2020). Morpholine as ubiquitous pharmacophore in medicinal chemistry: deep insight into the structure-activity relationship (SAR). Bioorg Chem.

[CR14] Le CD, Pham CT, Nguyen HH (2019). Zinc(II) {2}-metallacoronates and {2}-metallacryptates based on dipicolinoylbis(N, N-diethylthiourea): Structures and biological activities. Polyhedron.

[CR15] Lever S, Lydon J, Cutler C, Jurisson SS, McCleverty JA, Meyer TJ (2003). Comprehensive coordination chemistry II.

[CR16] Macrae CF, Sovago I, Cottrell SJ, Galek PTA, McCabe P, Pidcock E, Platings M, Shields GP, Stevens JS, Towler M, Wood PA (2020). Mercury 4.0: from visualization to analysis, design and prediction. J Appl Cryst.

[CR17] Maia PIS, Nguyen HH, Hagenbach A, Bergemann S, Gust R, Deflon VM, Abram U (2013). Rhenium mixed-ligand complexes with S, N, S-tridentate thiosemicarbazone/thiosemicarbazide ligands. Dalton Trans.

[CR18] Maia PIS, Deflon VM, Abram U (2014). Gold(III) complexes in medicinal chemistry. Future Med Chem.

[CR19] Marzo T, Cirri D, Gabbiani C, Gamberi T, Magherini F, Pratesi A, Guerri A, Biver T, Binacchi F, Stefanini M, Arcangeli A, Messori L (2017). Auranofin, Et_3_PAuCl, and Et_3_PAuI are highly cytotoxic on colorectal cancer cells: a chemical and biological study. ACS Med Chem Lett.

[CR20] Nelson BJB, Andersson JD, Wuest F, Spreckelmeyer S (2022). Good practices for ^68^Ga radiopharmaceutical production. EJNMMI Radiopharm Chem.

[CR21] Nguyen HH, Jegathesh JJ, Takiden A, Hauenstein D, Pham CT, Le CD, Abram U (2016). 2,6-Dipicolinoylbis(*N, *N-dialkylthioureas) as versatile building blocks for oligo- and polynuclear architectures. Dalton Trans.

[CR22] Pham CT, Nguyen HH, Hagenbach A, Abram U (2017). Iron(III) metallacryptand and metallacryptate assemblies derived from aroylbis(N, N-diethylthioureas). Inorg Chem.

[CR23] Pham CT, Nguyen TH, Trieu TN, Matsumoto K, Nguyen HH (2019). Syntheses, structures, and bioactivity evaluation of some transition metal complexes with aroylbis(N,N diethylthioureas) derived from natural compounds. Z Anorg Allg.

[CR24] Pham CT, Nguyen TH, Matsumoto K, Nguyen HH (2019). Cu^I^/Cu^II^ Complexes with dipicolinoylbis(N,N-diethylthiourea): structures, magnetism, and guest ion exchange. Eur J Inorg Chem.

[CR25] Pham CT, Jungfer MR, Abram U (2020). Indium(III) {2}-metallacryptates assembled from 2,6-dipicolinoyl-bis(N, N-diethylthiourea). New J Chem.

[CR26] Price EW, Orvig C (2014). Matching chelators to radiometals for radiopharmaceuticals. Chem Soc Rev.

[CR27] Reichert DE, Sewis LJ, Anderson CJ (1999). Metal complexes as diagnostic tools. Coord Chem Rev.

[CR28] Rodenstein A, Griebel J, Richter R, Kirmse R (2008). Synthese, Struktur und EPR-Untersuchungen von binuklearen Bis(N, N, N‴, N‴-tetraisobutyl-N′, N″-isophthaloylbis(thioureato))-Komplexen des CuII, NiII, ZnII, CdII und PdII. Z Anorg Allg Chem.

[CR29] Roesch F, Riss PJ (2010). The renaissance of the ^68^Ge/^68^Ga radionuclide generator initiates new developments in ^68^Ga radiopharmaceutical chemistry. Curr Top Med Chem.

[CR30] Rösch F (2013). Past, present and future of 68Ge/68Ga generators. Appl Radiat Isot.

[CR31] Salsi F, Portapilla GB, Simon S, Jungfer MR, Hagenbach A, de Albuquerque S, Abram U (2019). Effect of Fluorination on the structure and anti-trypanosoma cruzy activity of oxorhenium(V) complexes with S, N, S-tridentate thiosemicarbazones and benzoylthioureas: synthesis and structures of technetium(V) analogues. Inorg Chem.

[CR32] Schröder U, Beyer L, Sieler J (2000). Synthesis and X-ray structure of a new silver(I) coordination polymer assembled as one-dimensional chains. Inorg Chem Commun.

[CR33] Sheldrick G (2008). A short history of SHELX. Acta Crystallogr Sect A.

[CR34] Sheldrick G (2015). Crystal structure refinement with SHELXL. Acta Crystallogr Sect C.

[CR35] Sucena SF. Gold complexes and cages with aroylthioureas. *Doctoral *Thesis Freie Universität Berlin (2018). https://refubium.fu-berlin.de/handle/fub188/12274.

[CR36] Sucena SF, Pham TT, Hagenbach A, Pham CT, Abram U (2020). Structural diversity of alkaline earth centered gold(I) metallacoronates. Eur J Inorg Chem.

[CR37] Sucena SF, Demirer TI, Baitullina A, Hagenbach A, Grewe J, Spreckelmeyer S, März J, Barkleit A, da Maia PI, Nguyen H, Abram U (2023). Gold-based coronands as hosts for M^3+^ metal ions: ring size matters. Molecules.

[CR38] Uson R, Laguna A, Laguna M, Briggs DA, Murray HH, Fackler JP, Kaesz HD (1989). (Tetrahydrothiophene)gold(i) or gold(iii) complexes. Inorganic syntheses.

[CR39] Xie M, Zhu G, Hu Y, Gu H (2011). Conformations of morpholine in liquid and adsorbed on gold nanoparticles explored by Raman spectroscopy and theoretical calculations. J Phys Chem C.

[CR40] X-RED32, STOE & Cie GmbH: Darmstadt, Germany (2002).

[CR41] Yokoyama M, Ikuma T, Obara N, Togo H (1990). Synthesis of mesoionic triazoline nucleosides. J Chem Soc Perkin Trans.

