# Supplementary material for: Metallacages with 2,6-dipicolinoylbis(N,N-dialkylthioureas) as novel platforms in nuclear medicine for 68Ga, 177Lu and 198Au
Source: EJNMMI Radiopharm Chem. 2023 Nov 20;8:40. doi: 10.1186/s41181-023-00225-z (PMC10661681; doi:10.1186/s41181-023-00225-z)
Supplement: Supplementary file 1 — Additional file 1. Supplementary Information. [file 41181_2023_225_MOESM1_ESM.docx]

*Supplementary Information*

**Metallacages with 2,6-Dipicolinoylbis(*N,N*-dialkylthioureas) as novel platforms in nuclear medicine for ^68^Ga, ^177^Lu and ^198^Au**

Anna Baitullina,^a^ Guilhem Claude,^a^ Suelen F. Sucena,^a^ Eda Nisli,^b^ Cedric Scholz,^a^ Holger Amthauer,^b^ Winfried Brenner,^b^ Christopher Geppert,^c^ Christian Gorges,^c^  Ulrich Abram,^a^ Pedro Ivo da Silva Maia^d, *^ and Sarah Spreckelmeyer^b,^ *

**^a^** Institute of Chemistry and Biochemistry, Freie Universität Berlin, Fabeckstr. 34-36, D-14195 Berlin, Germany

**^b^** Charité - Universitätsmedizin Berlin, corporate member of Freie Universität Berlin, Humboldt-Universität zu Berlin, and Berlin Institute of Health, Department of Nuclear Medicine, Augustenburger Platz 1, 13353, Berlin, Germany

**^c^** Forschungsreaktor TRIGA Mainz, Johannes Gutenberg-Universität Mainz, Fritz-Strassmann-Weg 2, 55128 Mainz, Germany.

^d^ Núcleo de Desenvolvimento de Compostos Bioativos (NDCBio), Universidade Federal do Triângulo Mineiro, 38025-440, Uberaba, MG, Brazil.

*corresponding authors: [sarah.spreckelmeyer@charite.de](mailto:sarah.spreckelmeyer@charite.de), [pedro.maia@uftm.edu.br](mailto:pedro.maia@uftm.edu.br)

**Table of Contents**

**[Part 1: Crystallographic data](#_heading=h.gjdgxs)** S4

*[Figure S1.1](#_heading=h.3znysh7).* Ellipsoid representation of [La{Au(L^morph^-κS)}_3_]∙3MeOH∙1.5CH_2_Cl_2._ [S](#_heading=h.3znysh7)4

*[Figure S1](#_heading=h.2et92p0).2* Ellipsoid representation of [Lu{Au(L^morph^-κS)}_3_]∙MeOH∙2CH_2_Cl_2._ S5

*[Figure S1.3](#_heading=h.tyjcwt)* Ellipsoid representation of [Tb{Au(L^morph^-κS)}_3_]∙2CH_2_Cl_2_∙MeCN.S6

*[Figure S1.](#_heading=h.tyjcwt)4* Ellipsoid representation of [Y{Au(L^morph^-κS)}_3_]∙2CH_2_Cl_2_∙MeOH. S7

*[Table S1.](#_heading=h.tyjcwt)* Refinement data for the complexes S8-S9

*[Table](#_heading=h.tyjcwt) S2.* Selected bond lengths (Å) and angles (°) for the complexes S10-S11

**[PART 2 Spectroscopic and spectrometric data](#_heading=h.3dy6vkm)**  S12

*[Figure S2.1.](#_heading=h.4d34og8)* FTIR spectrum of the complex [La{Au(L^morph^-*κS*)}_3_] (**1**) (ATR, cm^-1^). S[12](#_heading=h.4d34og8)

*[Figure S2.2.](#_heading=h.4d34og8)* FTIR spectrum of the complex [Tb{Au(L^morph^-*κS*)}_3_] (**2**) (ATR, cm^-1^).  [S12](#_heading=h.4d34og8)

*[Figure S2.3.](#_heading=h.4d34og8)* FTIR spectrum of the complex [Lu{Au(L^morph^-*κS*)}_3_] (**3**) (ATR, cm^-1^). S[1](#_heading=h.4d34og8)3

*[Figure S2.4.](#_heading=h.4d34og8)* FTIR spectrum of the complex [Y{Au(L^morph^-*κS*)}_3_] (**4**) (ATR, cm^-1^). S13

*[Figure S2.5.](#_heading=h.4d34og8)* FTIR spectrum of the complex [Ga{Au(L^morph^-*κS*)}_2_] (**5**) (ATR, cm^-1^). S[1](#_heading=h.4d34og8)4

*[Figure S2.6.](#_heading=h.4d34og8)* ^1^H NMR spectrum of the complex [La{Au(L^morph^-*κS*)}_3_] (**1**) from CDCl_3_ solution (δ, ppm). S[1](#_heading=h.4d34og8)5

*[Figure S2.7.](#_heading=h.4d34og8)* ^1^H NMR spectrum of the complex [Lu{Au(L^morph^-*κS*)}_3_] (**3**) from CDCl_3_ solution (δ, ppm). S[15](#_heading=h.4d34og8)

*[Figure S2.8.](#_heading=h.4d34og8)* ^1^H NMR spectrum of the complex [Y{Au(L^morph^-*κS*)}_3_] (**4**) from CDCl_3_ solution (δ, ppm). S[1](#_heading=h.4d34og8)6

*Figure S2.9.* ESI^+^ MS of the complex [La{Au(L^morph^-*κS*)}_3_] (**1**) from MeCN solution. S17

*Figure S2.10.* ESI^+^ MS of the complex [Lu{Au(L^morph^-*κS*)}_3_] (**3**) from MeCN solution S18

*Figure S2.11.* ESI^+^ MS of the complex [Y{Au(L^morph^-*κS*)}_3_] (**4**) from MeCN solution. S18

*Figure S2.12.* ESI^+^ MS of the complex [Ga{Au(L^morph^-*κS*)}_2_] (**5**) from MeCN solution. S19

**[Part 3.](#_heading=h.2s8eyo1)**  **Radiolabeling data** [. S20](#_heading=h.2s8eyo1)

*[Figure S3.1.](#_heading=h.17dp8vu)* UV chromatograms of A) Free H_2_L^diethyl^; B) reaction mixture of H_2_L^diethyl^, [AuCl(THT)] and Ga(NO_3_)_3_ C) isolated cold complex [Ga{Au(L^diethyl^)}_2_]^+^. [S](#_heading=h.17dp8vu)21

*[Figure S3.2](#_heading=h.3rdcrjn)*[.](#_heading=h.3rdcrjn) ESI^+^ MS of [Ga{Au(L^diethyl^)}_2_] of the HPLC fraction at t_R_ = 9.8 min in water/acetonitrile.S22

*[Figure S3.3](#_heading=h.3rdcrjn)*[.](#_heading=h.3rdcrjn) Radio-chromatograms A) H_2_L^ethyl^, [AuCl(THT)] plus ^68^Ga in DMSO/Na-acetate B) starting from [Ga{Au(L^diethyl^)}_2_]^+^ and adding [^68^Ga]GaCl_3_; starting materials dissolved in DMSO; 90 °C 10 min.S23

*[Figure S3.4](#_heading=h.3rdcrjn)*[.](#_heading=h.3rdcrjn) UV Chromatograms A) Uncoordinated H_2_L^morph^ B) isolated cold complex [Ga{Au(L^morph^)}_2_]^+^. S24

*[Figure S3.](#_heading=h.3rdcrjn)5*[.](#_heading=h.3rdcrjn) iTLC-chromatograms of A) [^68^Ga][Ga{Au(L^morph^)}_2_]^+^ B) [^68^Ga][Ga{Au(L^morph^)}_2_]^+^ with human serum albumin.S25

*[Figure S3.](#_heading=h.3rdcrjn)6*[.](#_heading=h.3rdcrjn) Radio-Chromatogram of [^198^Au][Ga{Au(L^morph^)}_2_]^+^.S26

*[Figure S3.](#_heading=h.3rdcrjn)7*[.](#_heading=h.3rdcrjn) Radio-chromatogram of [^198^Au][Lu{Au(L^diethyl^)}_3_].S27

*[Figure S3.](#_heading=h.3rdcrjn)8*[.](#_heading=h.3rdcrjn) Radio-Chromatogram of [^177^Lu][Lu{Au(L^morph^)}_3_].S28

**[Part 1: Crystallographic data](#_heading=h.gjdgxs)**


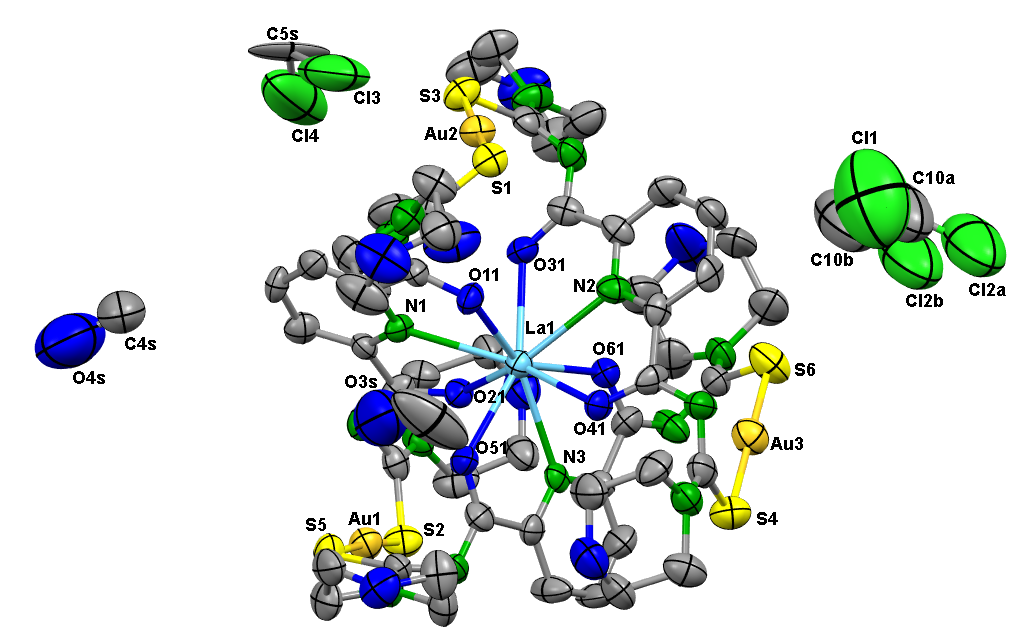


**Figure S1.1:** Ellipsoid representation of [La{Au(L^morph^-κS)}_3_]∙3MeOH∙1.5CH_2_Cl_2._ Thermal ellipsoids are at 50 % of probability. The hydrogen atoms have been omitted for clarity.


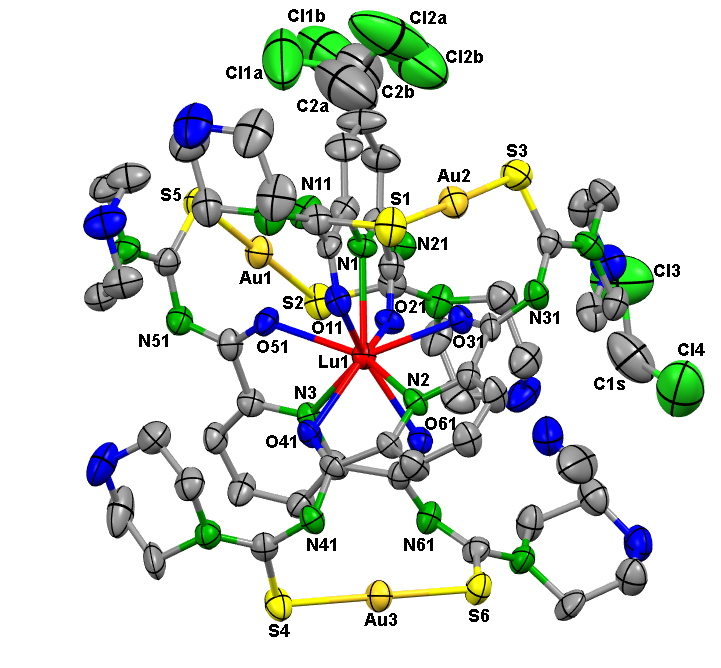


**Figure S1.2:** Ellipsoid representation of [Lu{Au(L^morph^-κS)}_3_]∙MeOH∙2CH_2_Cl_2._ Thermal ellipsoids are at 50 % of probability. The hydrogen atoms have been omitted for clarity.


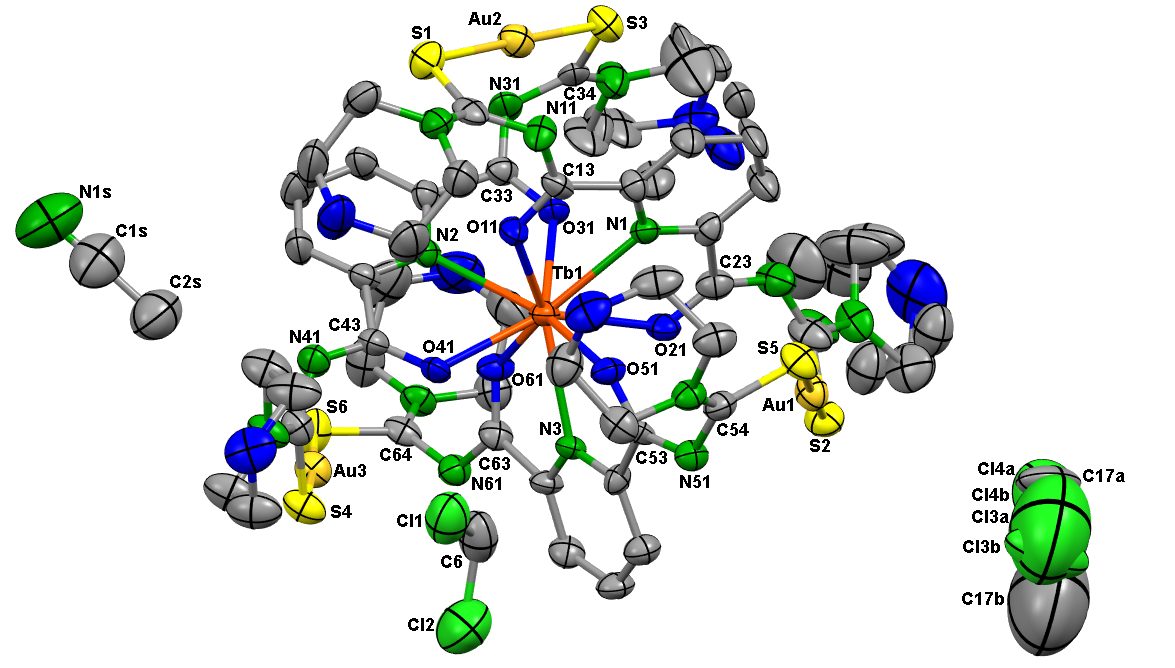


**Figure S1.3:** Ellipsoid representation of [Tb{Au(L^morph^-κS)}_3_]∙2CH_2_Cl_2_∙MeCN._._ Thermal ellipsoids are at 50 % of probability. The hydrogen atoms have been omitted for clarity.


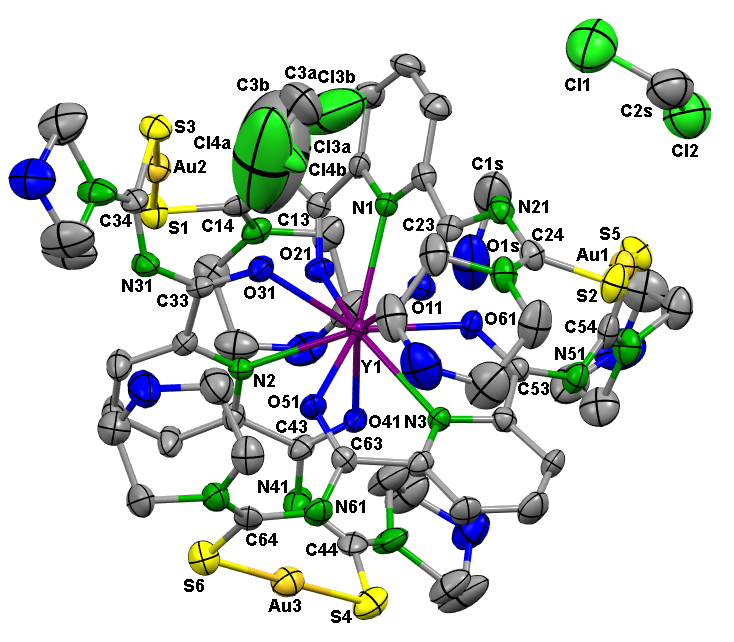


**Figure S1.4:** Ellipsoid representation of [Y{Au(L^morph^-κS)}_3_]∙2CH_2_Cl_2_∙MeOH. Thermal ellipsoids are at 50 % of probability. The hydrogen atoms have been omitted for clarity.

**Table S1.** Refinement data for the complexes [La{Au(L^morph^-κS)}3]∙3MeOH∙1.5CH_2_Cl_2_ (**1**), [Tb{Au(L^morph^-κS)}3]∙2CH_2_Cl_2_∙MeCN (**2**), [Lu{Au(L^morph^-κS)}3]∙MeOH∙2CH_2_Cl_2._ (**3**) and [Y{Au(L^morph^-κS)}3]∙2CH_2_Cl_2_∙MeOH. (**4**).

|  | **1** | **2** | **3** | **4** |
| --- | --- | --- | --- | --- |
| Formula | C_55.5_H_72_Au_3_Cl_3_LaN_15_O_15_S_6_ | C_55_H_64_Au_3_Cl_4_N_16_O_12_S_6_Tb | C_54_H_57_Au_3_Cl_4_LuN_15_O_13_S_6_ | C_54_H_63_Au_3_Cl_4_N_15_O_13_S_6_Y |
| Fw | 2217.80 | 2225.20 | 2224.17 | 2144.16 |
| *T* (K) | 200(2) | 200(2) | 200(2) | 200(2) |
| Crystal system | Monoclinic | Triclinic | Triclinic | Triclinic |
| Space group | *P*2_1_/*n* | *P* $\bar{1}$ | *P* $\bar{1}$ | *P* $\bar{1}$ |
| *a* (Å) | 14.2136(9) | 12.043(4) | 12.0475(11) | 12.212(6) |
| *b* (Å) | 12.1372(5) | 12.644(7) | 12.5458(14) | 12.609(6) |
| *c* (Å) | 43.783(3) | 24.329(8) | 24.4367(12) | 23.88(2) |
| *α* (°) |  | 84.60(4) | 84.98 | 86.70(6) |
| *β* (°) | 95.963(5) | 79.46(3) | 76.505(6) | 81.71(6) |
| *γ* (°) |  | 79.32(4) | 80.64 | 77.81(4) |
| V (Å^3^) | 7512.3(8) | 3572(3) | 3538.9(5) | 3555(4) |
| *Z* | 4 | 2 | 2 | 2 |
| *ρ*_calcd_ (g∙cm^–3^) | 1.961 | 2.069 | 2.087 | 2.003 |
| *μ* (mm^–1^) | 6.740 | 7.513 | 7.978 | 7.375 |
| Reflections Collected | 44722 | 30263 | 31403 | 29247 |
| Reflections unique/ R_int_ | 14573/0.1304 | 13951/0.0562 | 13582/0.0999 | 13912/0.0563 |
| Data/restraints/param. | 14573 / 26 / 906 | 13951 / 32 / 960 | 13582 / 15 / 884 | 13912 / 41 / 896 |
| Absorption correction | Integration | Integration | Integration | Integration |
| Max/min. transmission | 0.4587 0.2519 | 0.4351/0.2195 | 0.1977/0.0324 | 0.3650/0.0971 |
| R_1_ [*I* > 2*σ*(*I*)] | 0.0622 | 0.0384 | 0.0521 | 0.0366 |
| *wR*_2_ [*I* > 2σ(*I*)] | 0.1176 | 0.0580 | 0.1222 | 0.0721 |
| GOF on F^2^, *S* | 0.872 | 0.873 | 0.948 | 0.935 |
| CCDC number | 2258607 | 2258608 | 2258609 | 2258610 |

**Table S2.** Selected bond lengths (Å) and angles (°) for the complexes [La{Au(L^morph^-κS)}3]∙3MeOH∙1.5CH_2_Cl_2_ (**1**), [Tb{Au(L^morph^-κS)}3]∙2CH_2_Cl_2_∙MeCN (**2**), [Lu{Au(L^morph^-κS)}3]∙MeOH∙2CH_2_Cl_2._ (**3**) and [Y{Au(L^morph^-κS)}3]∙2CH_2_Cl_2_∙MeOH. (**4**).

|  | **1** | **2** | **3** | **4** |
| --- | --- | --- | --- | --- |
| **Bond lengths (Å)** |  |  |  |  |
| Au1–S2 | 2.288(4) | 2.290(2) | 2.280(3) | 2.283(2) |
| Au1–S5 | 2.287(4) | 2.297(2) | 2.286(3) | 2.280(2) |
| Au2–S1 | 2.284(4) | 2.288(2) | 2.268(3) | 2.288(2) |
| Au2–S3 | 2.286(4) | 2.297(2) | 2.284(3) | 2.286(2) |
| Au3–S4 | 2.277(4) | 2.278(2) | 2.279(3) | 2.296(3) |
| Au3–S6 | 2.285(4) | 2.275(2) | 2.279(3) | 2.293(3) |
| M^III^–N1 | 2.660(10) | 2.521(5) | 2.433(8) | 2.502(5) |
| M^III^–N2 | 2.662(11) | 2.490(5) | 2.451(8) | 2.490(5) |
| M^III^ –N3 | 2.653(9) | 2.526(5) | 2.427(6) | 2.492(4) |
| M^III^–O11 | 2.575(8) | 2.422(4) | 2.408(6) | 2.366(4) |
| M^III^–O21 | 2.452(9) | 2.479(5) | 2.333(6) | 2.366(4) |
| M^III^–O31 | 2.589(8) | 2.373(5) | 2.310(6) | 2.458(4) |
| M^III^–O41 | 2.491(9) | 2.460(4) | 2.342(6) | 2.373(4) |
| M^III^–O51 | 2.547(8) | 2.365(5) | 2.315(7) | 2.450(4) |
| M^III^–O61 | 2.508(8) | 2.372(4) | 2.406(6) | 2.366(4) |
| C13–O11 | 1.276(14) | 1.262(7) | 1.239(12) | 1.262(7) |
| C23–O21 | 1.302(16) | 1.245(8) | 1.262(12) | 1.278(7) |
| C33–O31 | 1.260(15) | 1.250(8) | 1.279(11) | 1.242(7) |
| C43–O41 | 1.281(15) | 1.253(8) | 1.287(11) | 1.263(7) |
| C53–O51 | 1.248(14) | 1.275(8) | 1.280(11) | 1.264(6) |
| C63–O61 | 1.245(14) | 1.264(8) | 1.279(10) | 1.265(6) |
| C14–S1 | 1.755(14) | 1.738(8) | 1.761(12) | 1.739(6) |
| C24–S2 | 1.733(13) | 1.740(9) | 1.740(11) | 1.733(6) |
| C34–S3 | 1.766(15) | 1.716(7) | 1.726(11) | 1.740(6) |
| C44–S4 | 1.727(16) | 1.735(8) | 1.721(10) | 1.721(7) |
| C54–S5 | 1.751(16) | 1.727(8) | 1.731(11) | 1.739(7) |
| C64–S6 | 1.715(15) | 1.722(7) | 1.742(11) | 1.731(7) |
| **Bond angles (°)** |  |  |  |  |
| S(2)-AU1-S(5) | 175.95(13) | 176.10(8) | 179.56(11) | 176.95(6) |
| S(1)-AU2-S(3) | 175.04(13) | 178.98(8) | 178.63(11) | 176.63(6) |
| S(6)-AU3-S(4) | 177.64(16) | 177.72(9) | 177.57(10) | 178.62(7) |
| O(21)-M^III^-O(41) | 142.6(3) | 149.30(16) | 140.4(2) | 141.47(13) |
| O(21)-M^III^-O(61) | 78.8(3) | 84.75(16) | 78.2(2) | 83.73(15) |
| O(41)-M^III^-O(61) | 85.6(3) | 81.13(16) | 81.6(2) | 88.22(15) |
| N(3)-M^III^-N(1) | 122.2(3) | 118.98(17) | 117.9(2) | 123.33(15) |
| N(2)-M^III^-N(1) | 124.4(3) | 115.67(18) | 119.8(2) | 119.86(14) |
| N(2)-M^III^-N(3) | 113.2(3) | 125.28(17) | 122.2(2) | 116.80(15) |
| O(31)-M^III^-N(3) | 136.7(3) | 133.28(16) | 139.2(2) | 134.59(15) |
| O(11)-M^III^-N(3) | 146.2(3) | 137.96(17) | 135.6(2) | 142.44(15) |
| O(61)-M^III^-N(1) | 132.7(3) | 141.78(17) | 138.0(3) | 136.78(16) |
| O(41)-M^III^-N(1) | 141.6(3) | 136.80(15) | 140.2(3) | 134.89(15) |
| O(51)-M^III^-N(2) | 135.9(3) | 141.94(17) | 134.4(3) | 136.90(15) |
| O(21)-M^III^-N(2) | 139.9(3) | 135.36(17) | 137.4(3) | 143.08(15) |
| O(11)-M^III^-N(1) | 60.2(3) | 64.19(16) | 64.9(2) | 64.05(15) |
| O(21)-M^III^-N(1) | 61.8(3) | 63.74(16) | 65.7(2) | 64.45(15) |
| O(31)-M^III^-N(2) | 61.3(3) | 65.37(17) | 66.0(2) | 64.13(15) |
| O(41)-M^III^-N(2) | 61.4(3) | 64.24(17) | 65.4(2) | 64.94(14) |
| C(13)-O(11)-M^III^ | 124.3(8) | 123.8(4) | 122.5(6) | 125.5(4) |
| C(33)-O(31)-M^III^ | 124.4(8) | 124.1(4) | 123.9(6) | 123.2(4) |
| C(53)-O(51)-M^III^ | 124.4(8) | 126.2(6) | 124.4(6) | 122.2(4) |

**PART 2. Spectroscopic and spectrometric data**




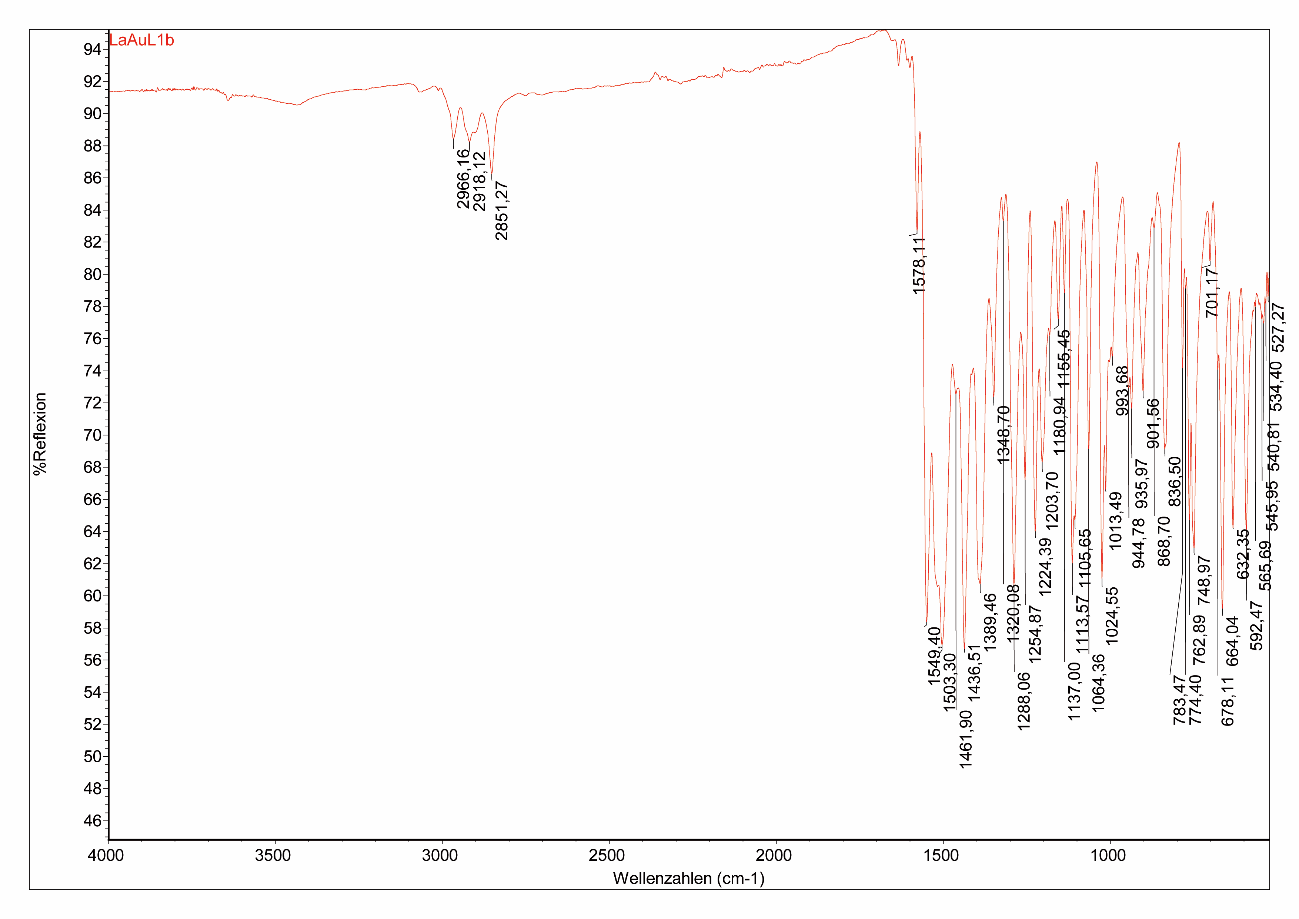


**Figure S2.1.** FTIR spectrum of the complex [La{Au(L^morph^-*κS*)}_3_] (**1**) (ATR, cm^-1^).




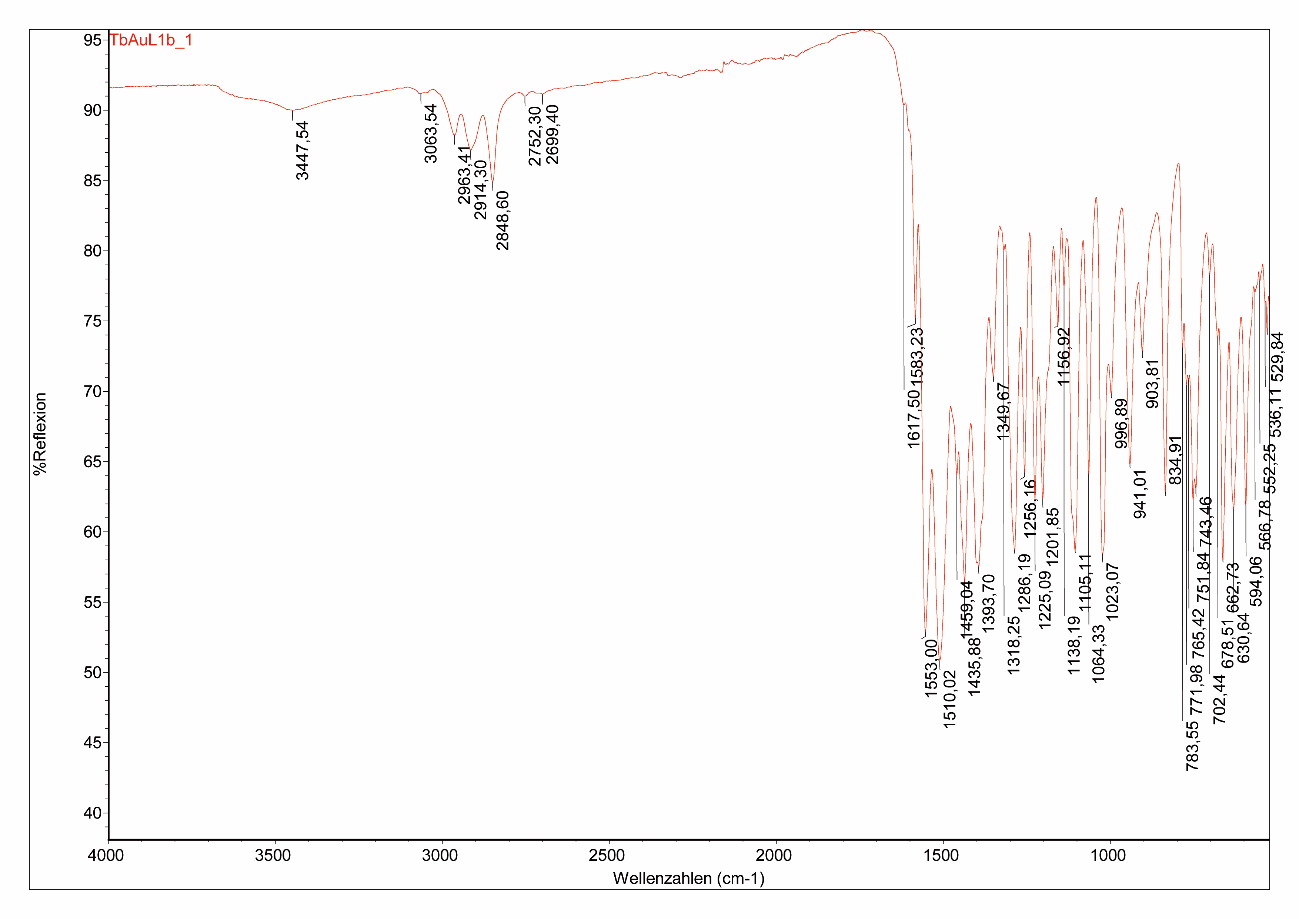


**Figure S2.2.** FTIR spectrum of the complex [Tb{Au(L^morph^-*κS*)}_3_] (**2**) (ATR, cm^-1^).



**
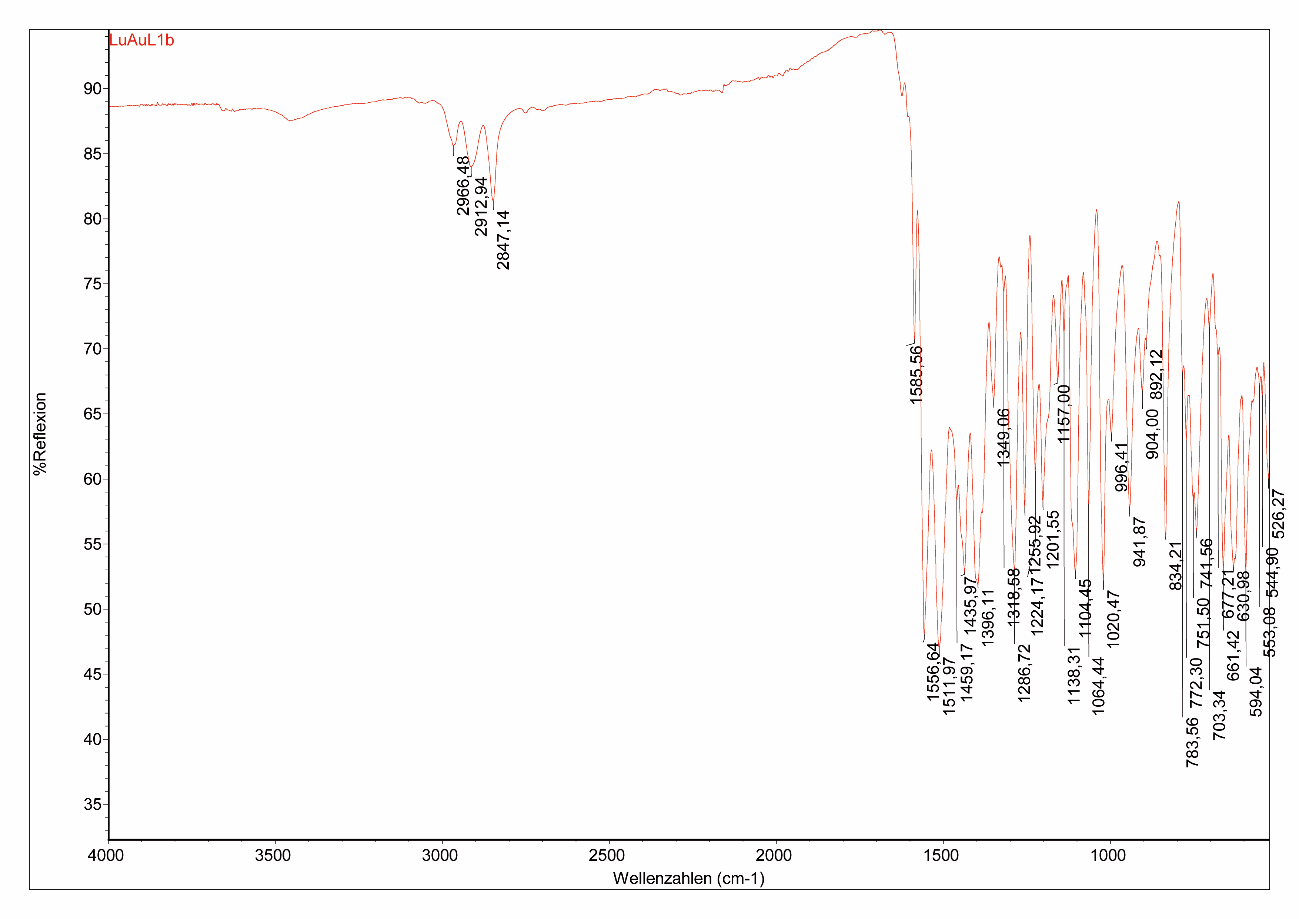
**

**Figure S2.3.** FTIR spectrum of the complex [Lu{Au(L^morph^-*κS*)}_3_] (**3**) (ATR, cm^-1^).




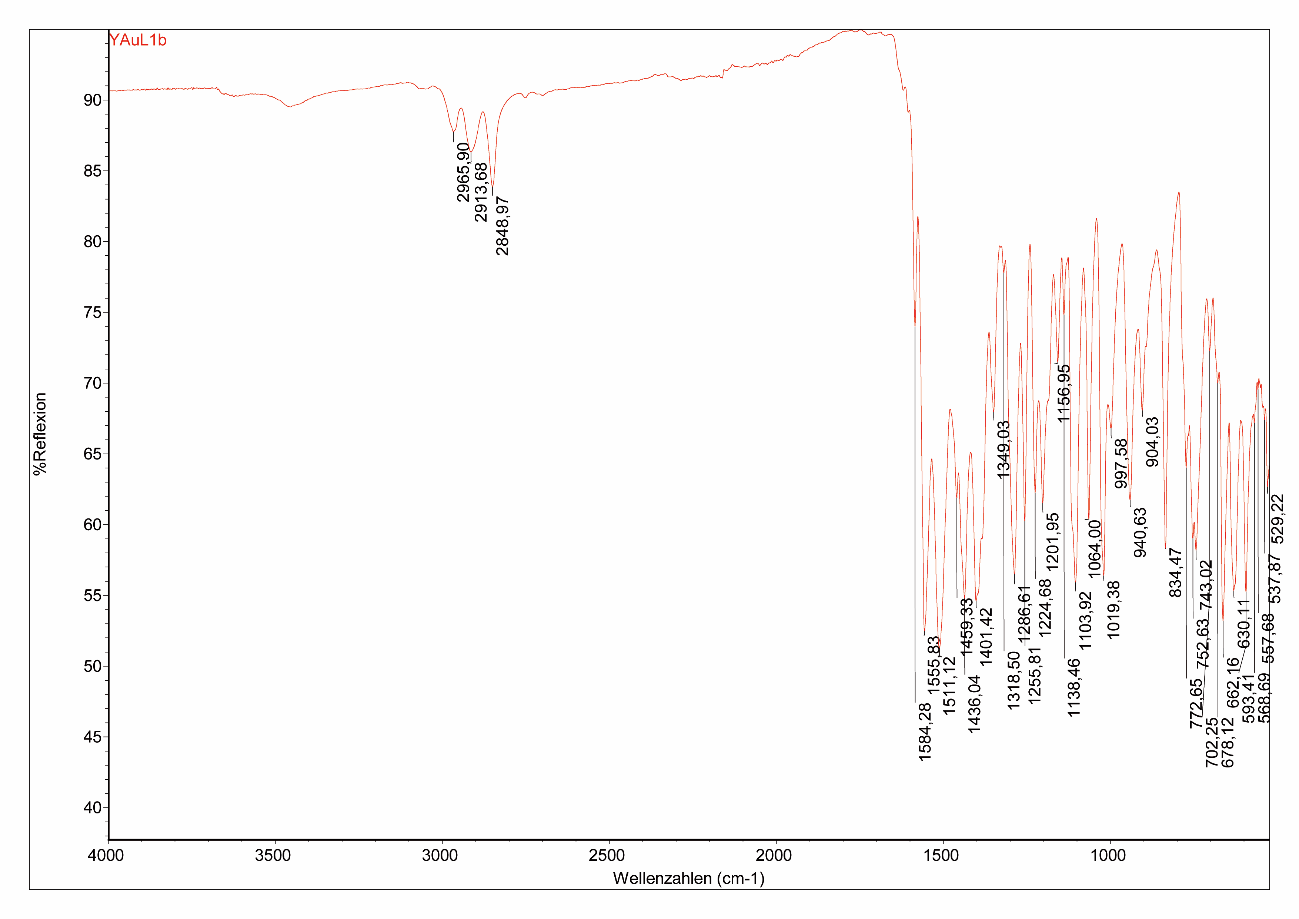


**Figure S2.4.** FTIR spectrum of the complex [Y{Au(L^morph^-*κS*)}_3_] (**4**) (ATR, cm^-1^).




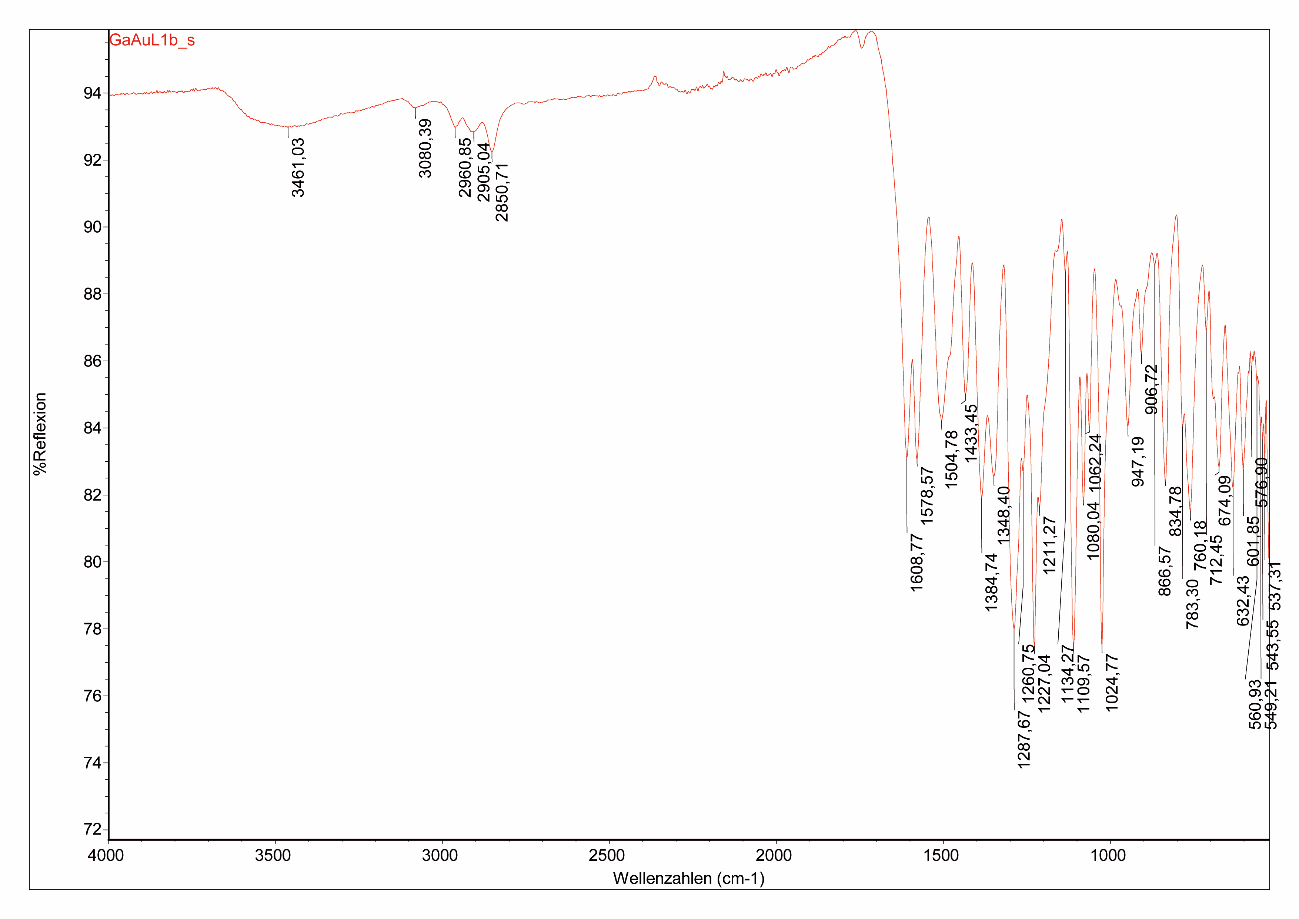


**Figure S2.5.** FTIR spectrum of the complex [Ga{Au(L^morph^-*κS*)}_3_](NO_3_) (**5**) (ATR, cm^-1^).

**NMR Spectra**




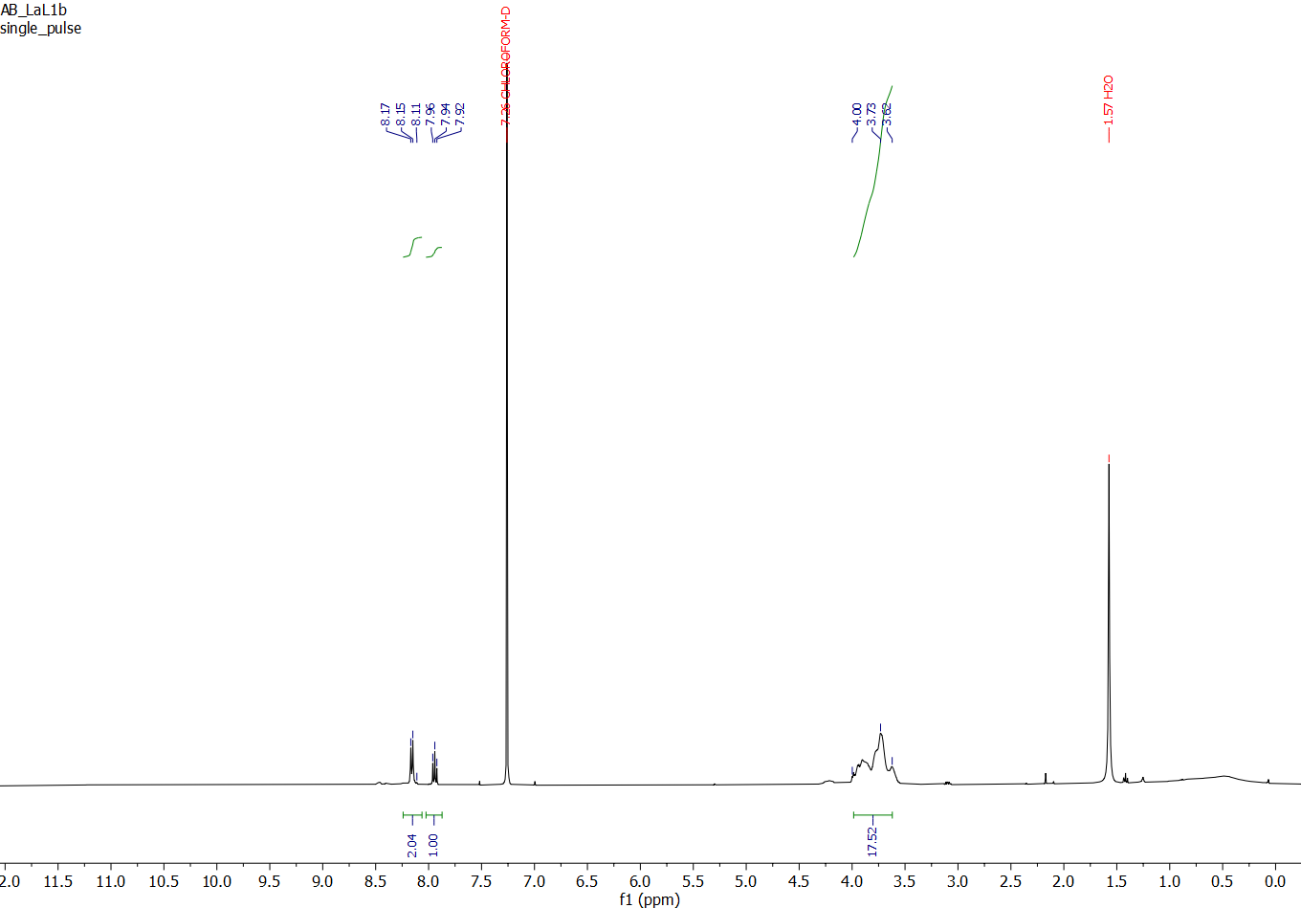


Py

Morph

**Figure S2.6.** ^1^H NMR spectrum of the complex [La{Au(L^morph^-*κS*)}_3_] (**1**) from CDCl_3_ solution (δ, ppm).




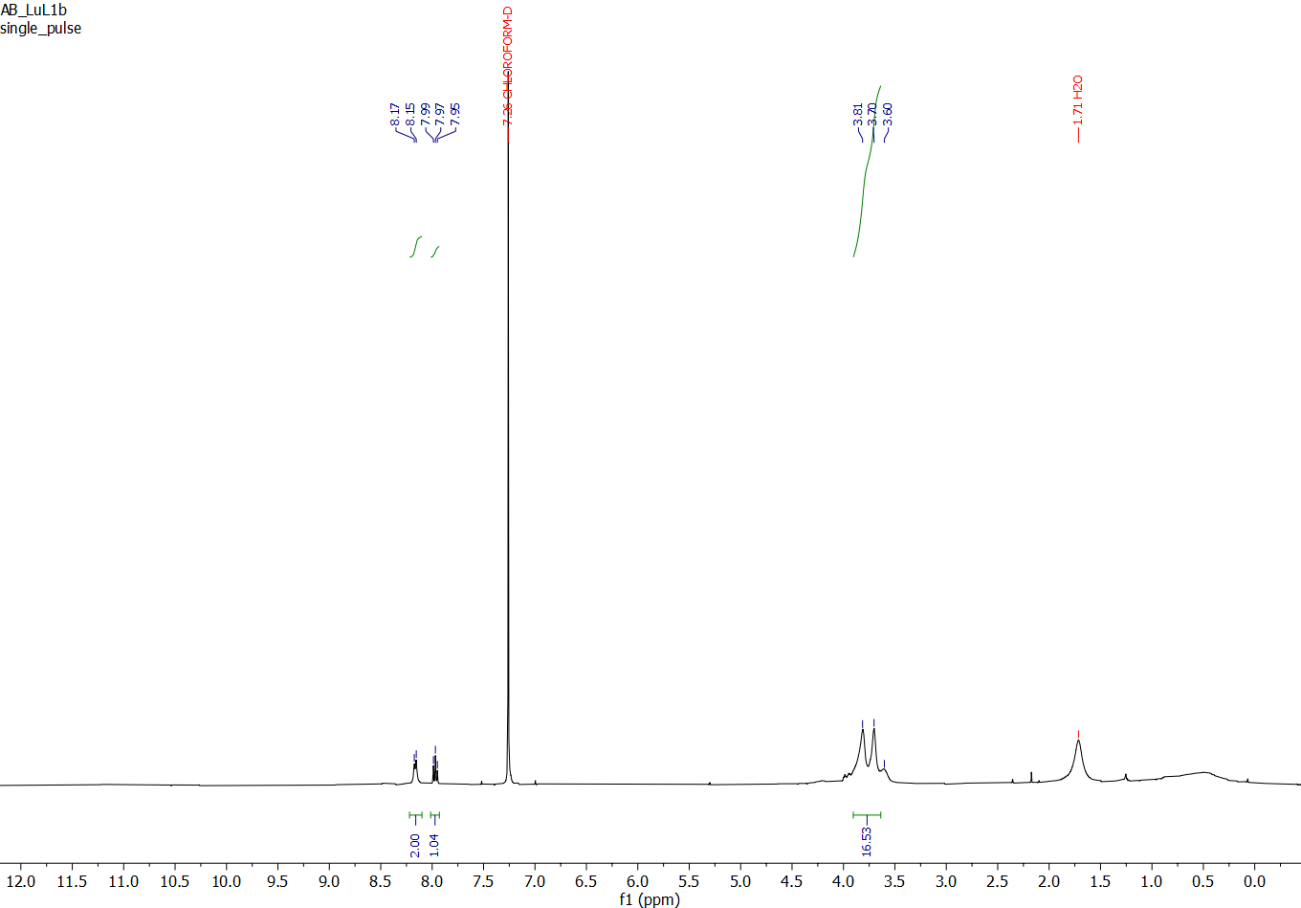


Py

Morph

**Figure S2.7.** ^1^H NMR spectrum of the complex [Lu{Au(L^morph^-*κS*)}_3_] (**3**) from CDCl_3_ solution (δ, ppm).



**
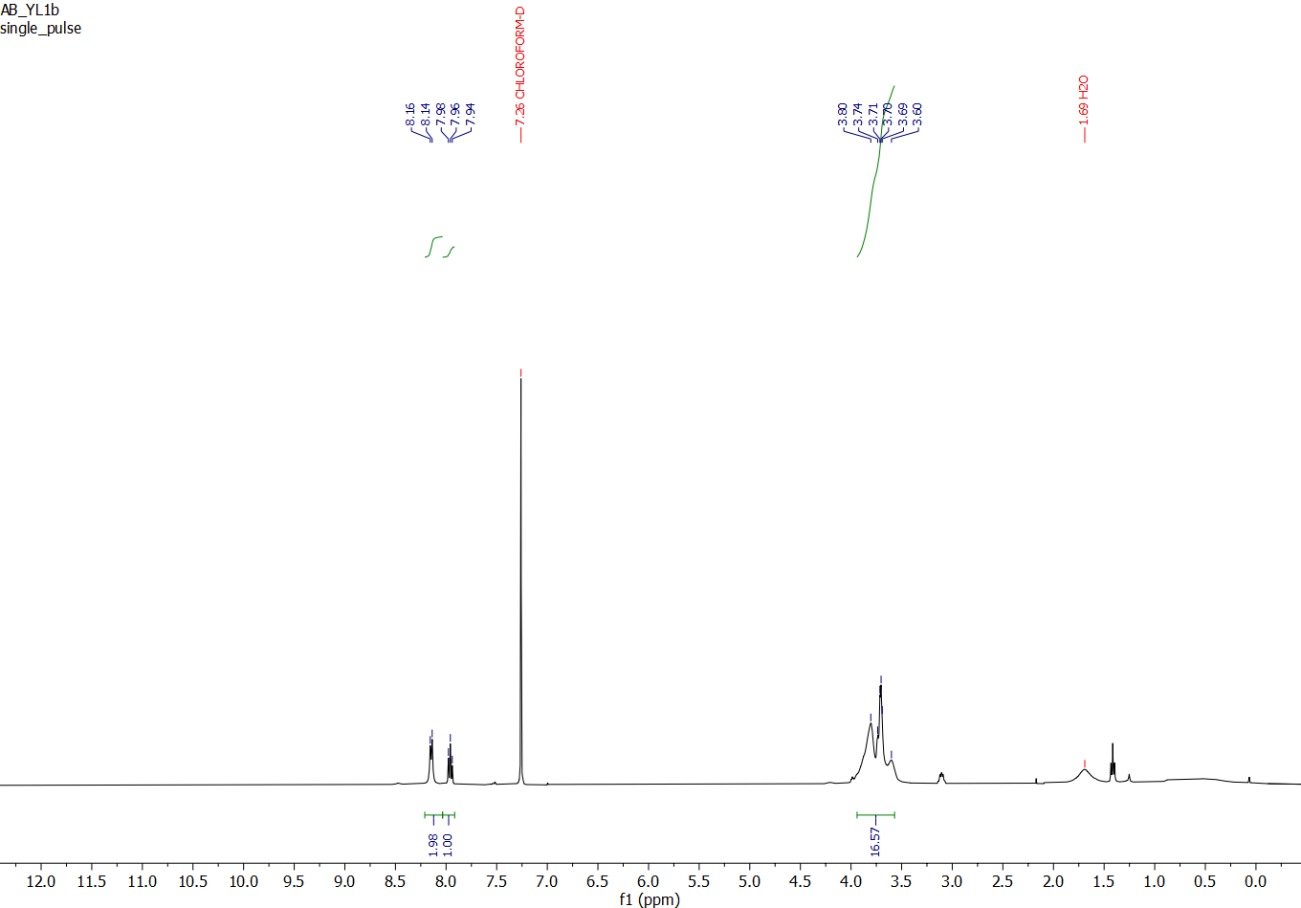
**

Py

Morph

**Figure S2.8.** ^1^H NMR spectrum of the complex [Y{Au(L^morph^-*κS*)}_3_] (**4**) from CDCl_3_ solution (δ, ppm).

**MS spectra**


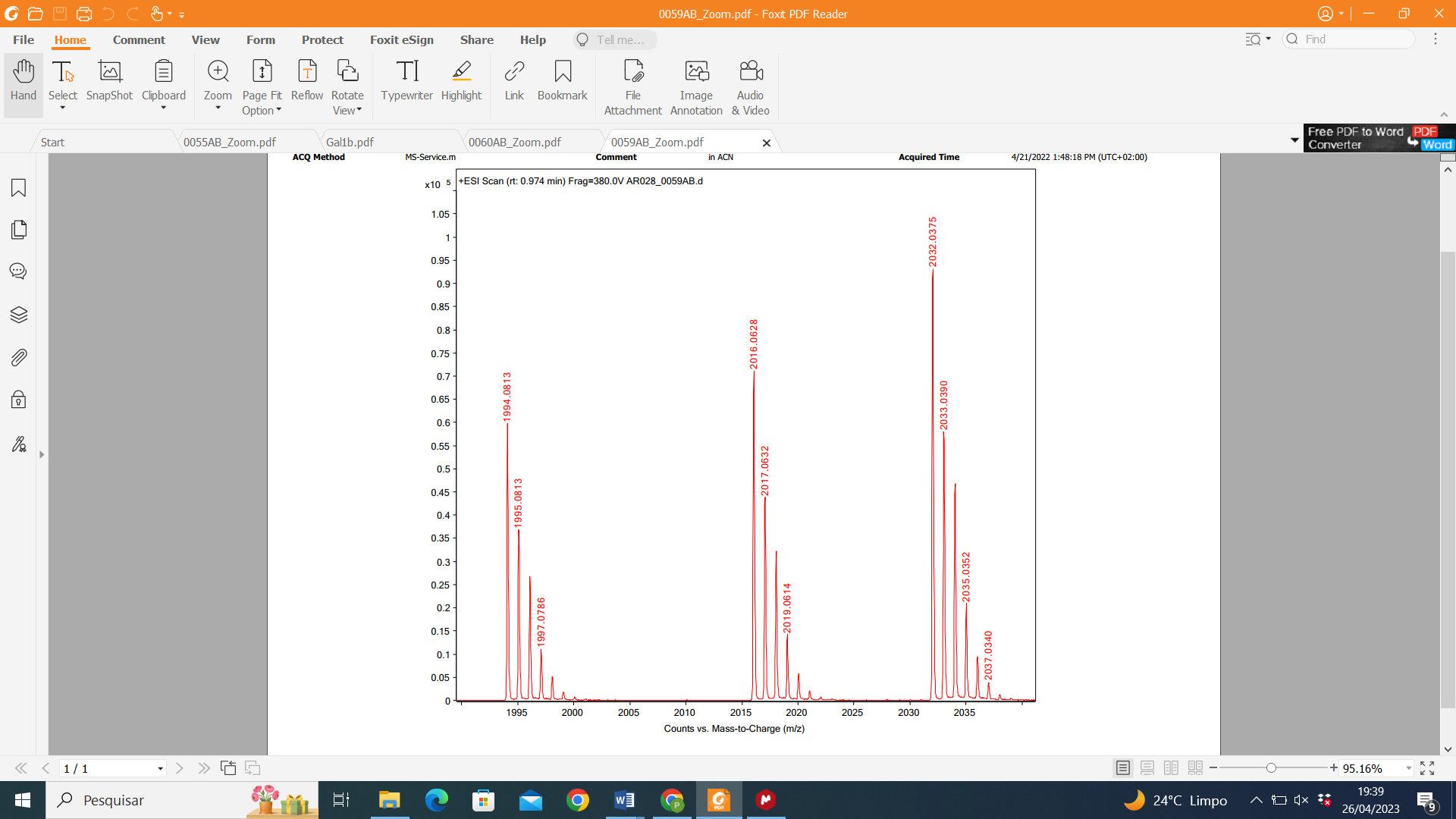

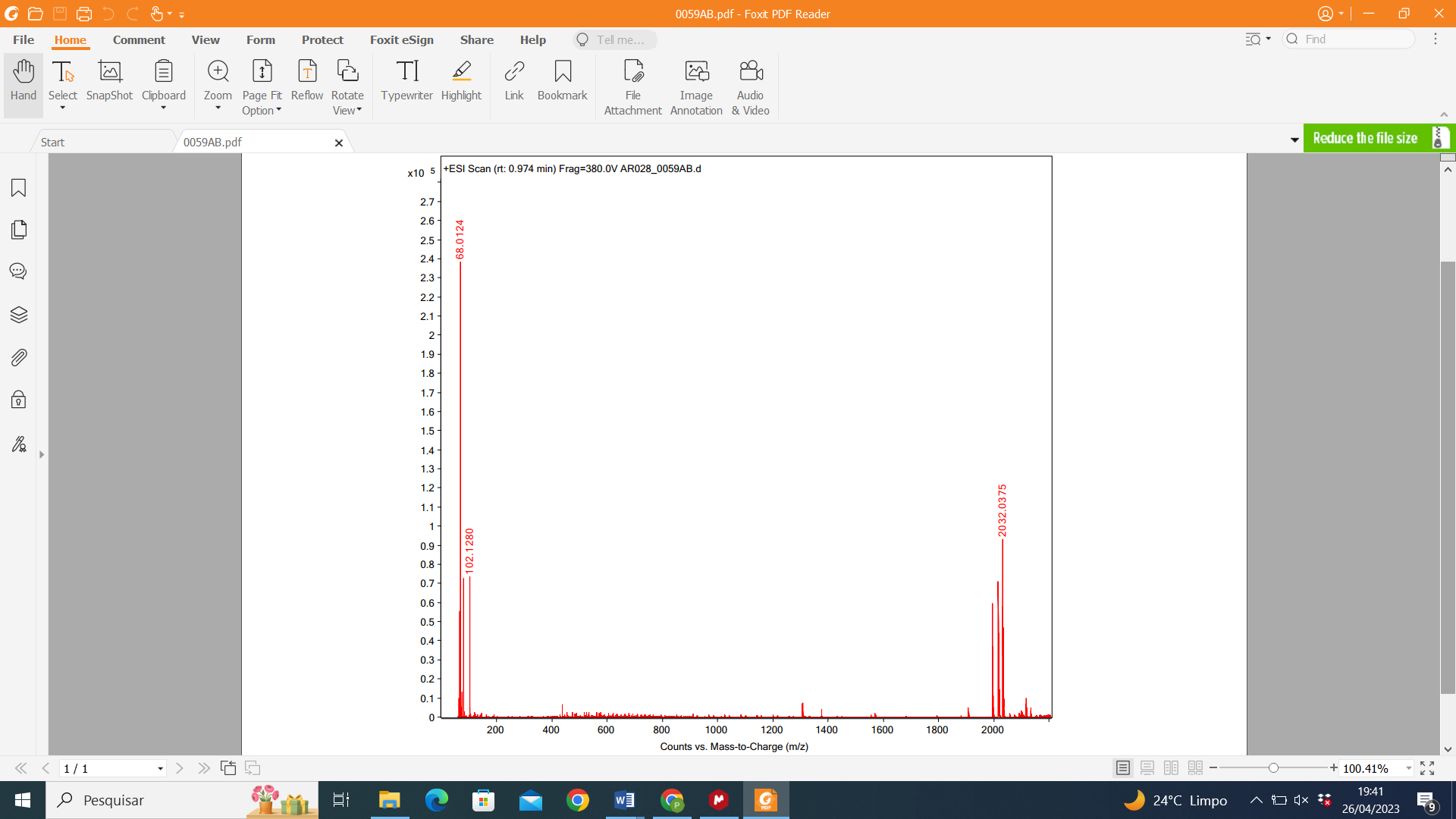


[M+H]^+^

(calcd. 1994.0729)

[M+Na]^+^

(calcd. 2016.0588)

[M+K]^+^

(calcd. 2032.0332)

**Figure S2.9.** ESI^+^ MS of the complex [La{Au(L^morph^-*κS*)}_3_] (**1**) from MeCN solution.


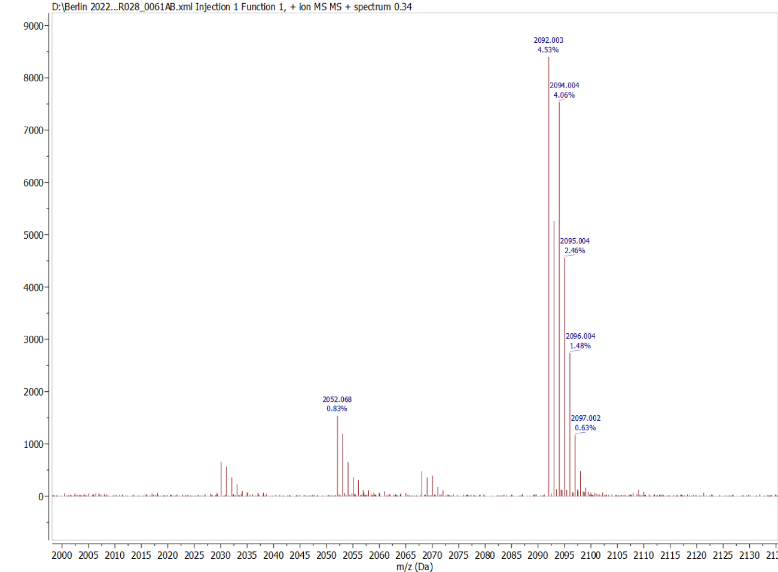

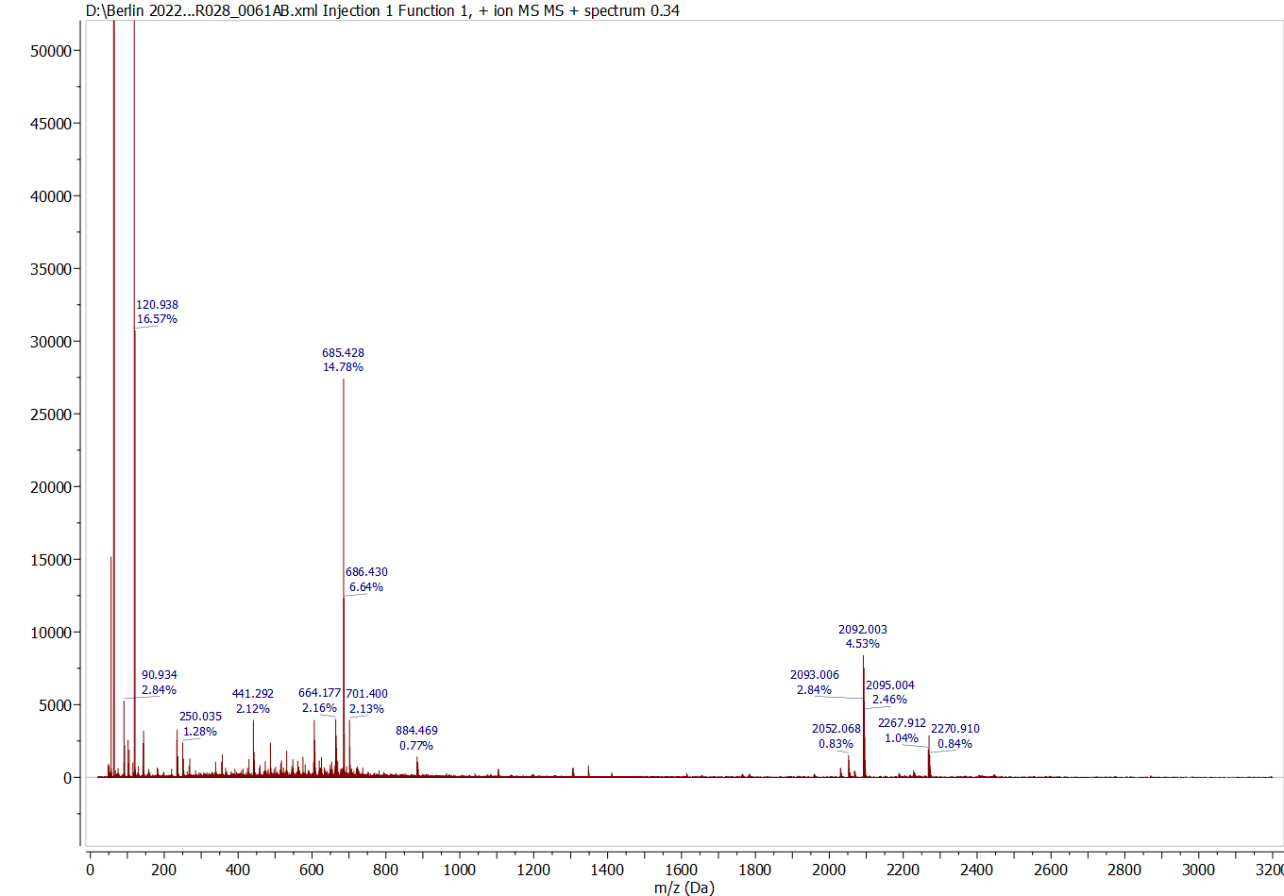


[M+Na+MeCN]^+^

(calcd. 2093.120)

[M+H]^+^

(calcd. 2030.112)

**Figure S2.10.** ESI^+^ MS of the complex [Lu{Au(L^morph^-*κS*)}_3_] (**3**) from MeCN solution.
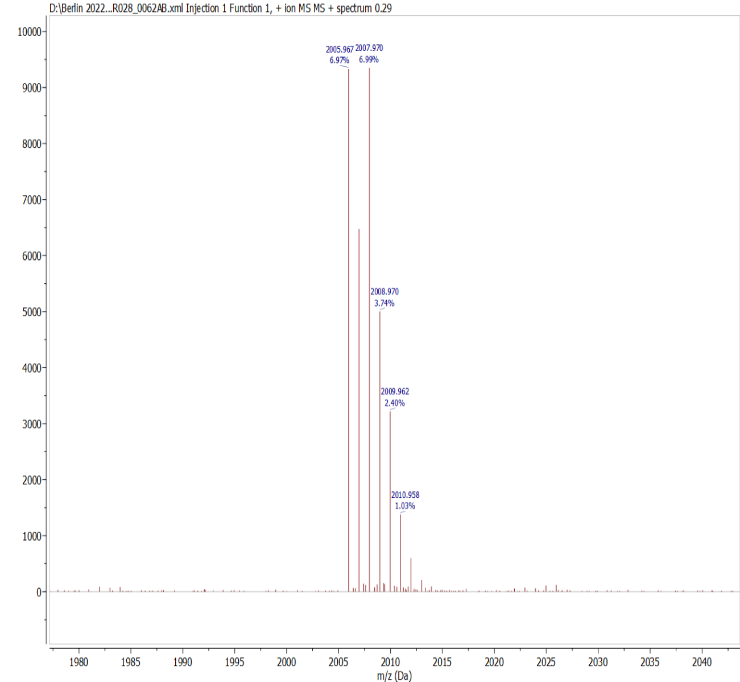

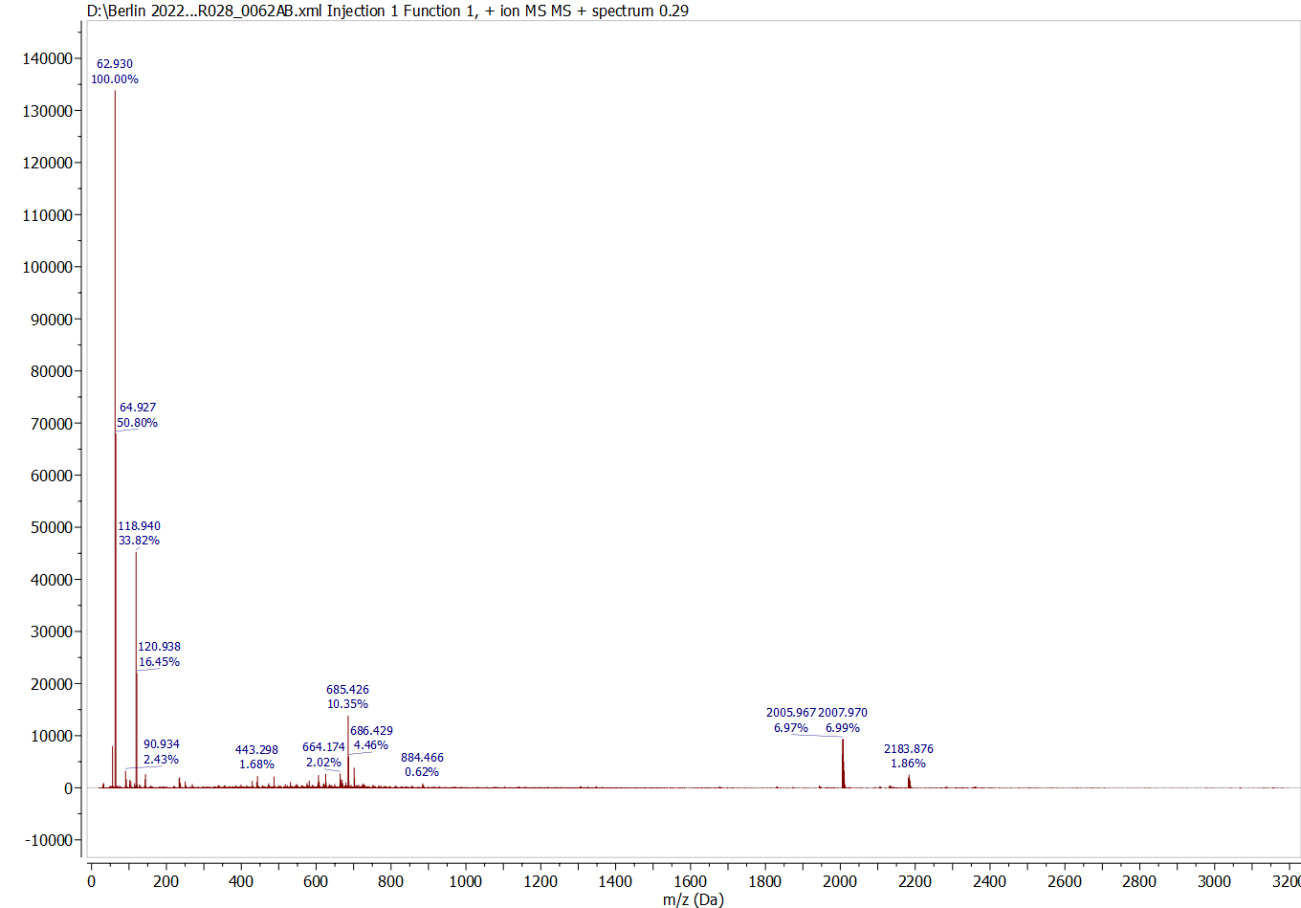


[M+Na+MeCN]^+^

(calcd. calcd. 2007.085)

**Figure S2.11.** ESI^+^ MS of the complex [Y{Au(L^morph^-*κS*)}_3_] (**4**) from MeCN solution.


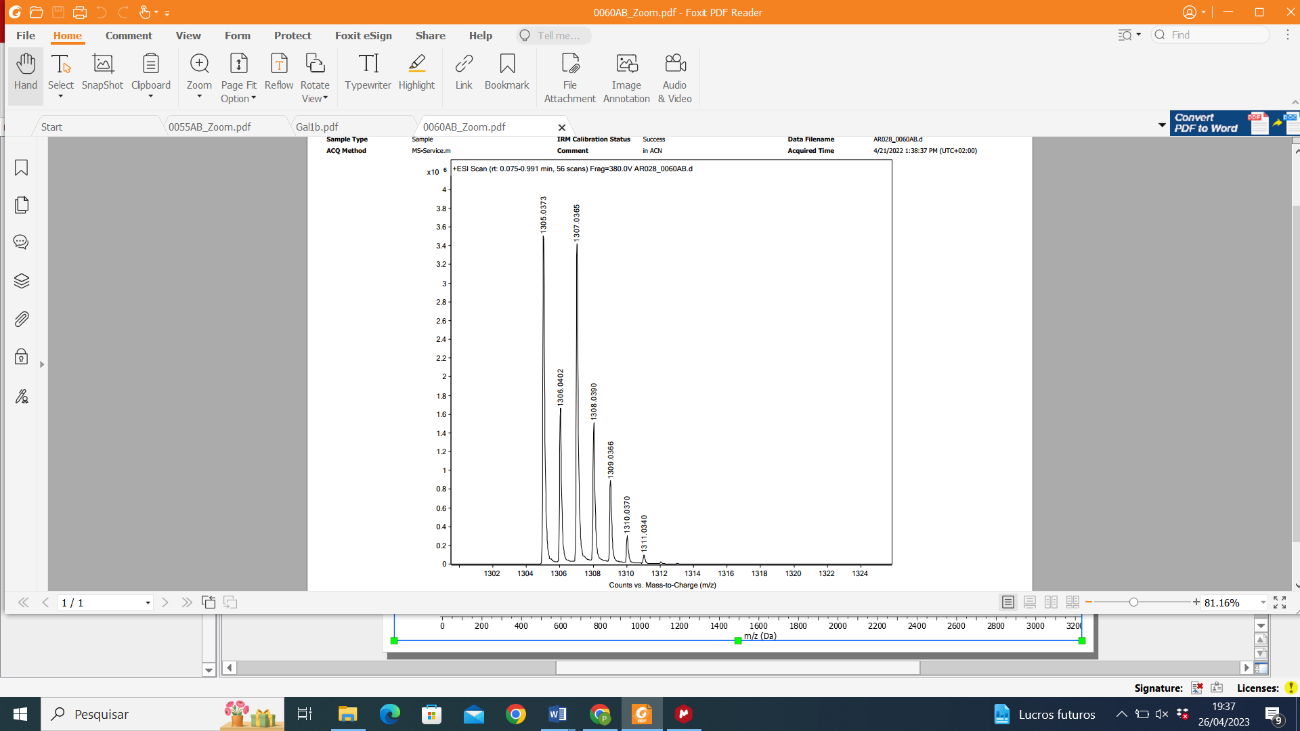

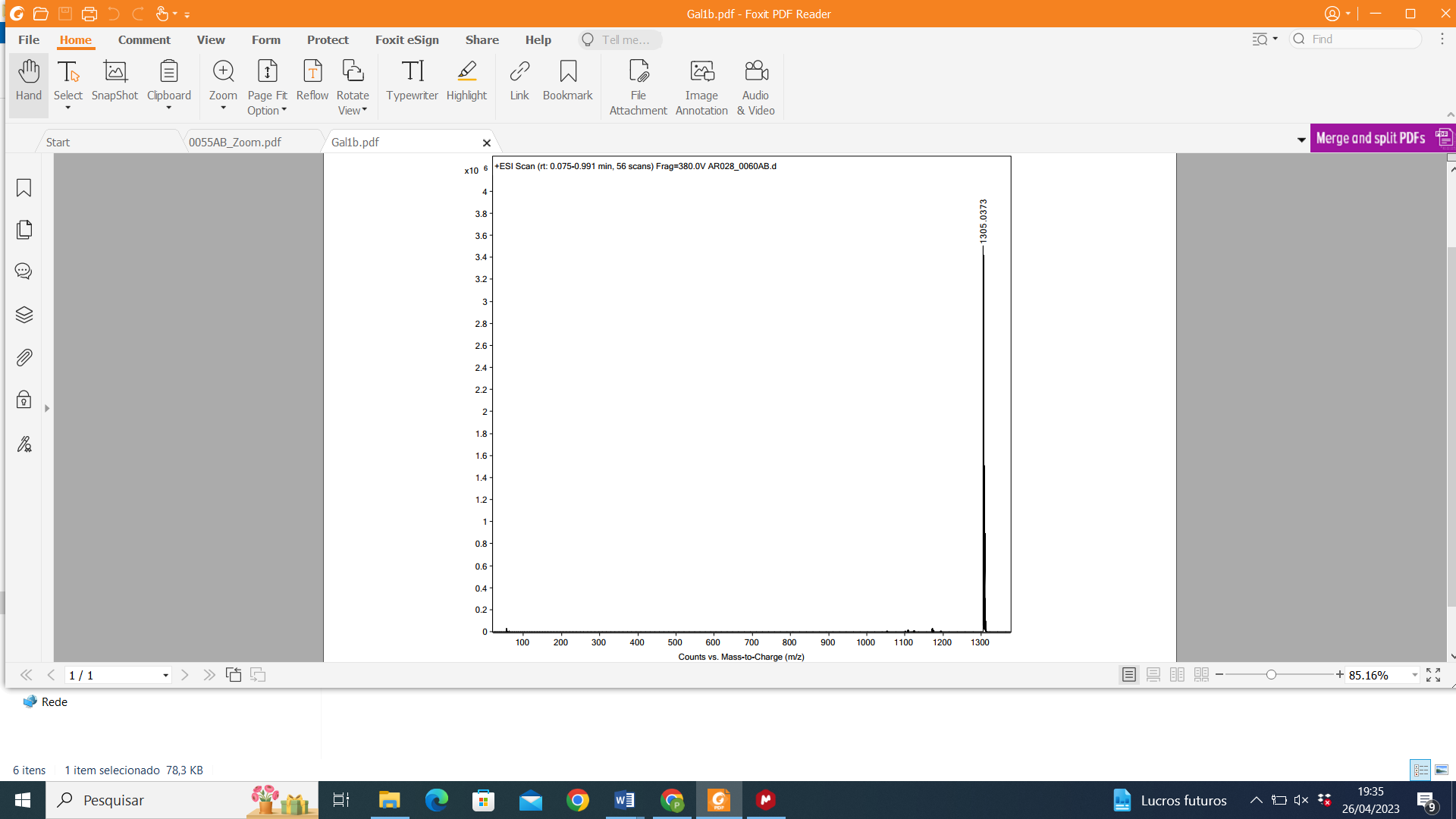


[M]^+^

(calcd. 1305.0338)

**Figure S2.12.** ESI^+^ MS of the complex [Ga{Au(L^morph^-*κS*)}_3_](NO_3_) (**5**) from MeCN solution.

**PART 3. Radiolabeling data**

**Figure S3.1** shows the UV chromatograms of the free H_2_L^diethyl^ ligand (**Figure S3.1a**). The free H_2_L^diethyl^ has a retention time of t_R_ = 9.58 min. The complexation reaction mixture for formation of the non-radioactive cationic complex [Ga{Au(L^diethyl^)}_2_]^+^ is shown in **Figure S3.1b**. Here, a peak at t_R_ = 9.63 min (free ligand), one at t_R_ = 10.88 min and two very close peaks at t_R_ = 13.48 min (metallacage) have been observed. The appearance of the peak at t_R_ = 10.88 in **Fig. S3.1b** is coherent with the formation of an intermediate compatible with a neutral complex with only one gold atom [Ga{Au(L^diethyl^)_2_}] (*m/z* calcd for [M+H]^+^ =1053.159), as indicated by the ESI-MS **Fig S3.2** (*m/z* found for [M+H]^+^ =1053.152). **Figure S3.1c** presents the UV chromatogram of the isolated complex [Ga{Au(L^diethyl^)}_2_]^+^ which has a retention time of 13.44 min.

A very similar profile is observed in the experiments with H_2_L^morph^ (**Figure S3.3**) with a retention time for the free H_2_L^morph^ of 7.78 min; Retention time of the isolated complex [Ga{Au(L^morph^)}_2_]^+^  is 9.53-9.98 min (two very close peaks); the retention times of the non-radioactive reaction can be attributed as follows: t_R_ =7.63 min (free ligand), t_R_ = 8.34 min (intermediate) and two very close peaks at t_R_ = 10.00 min (complex).

**
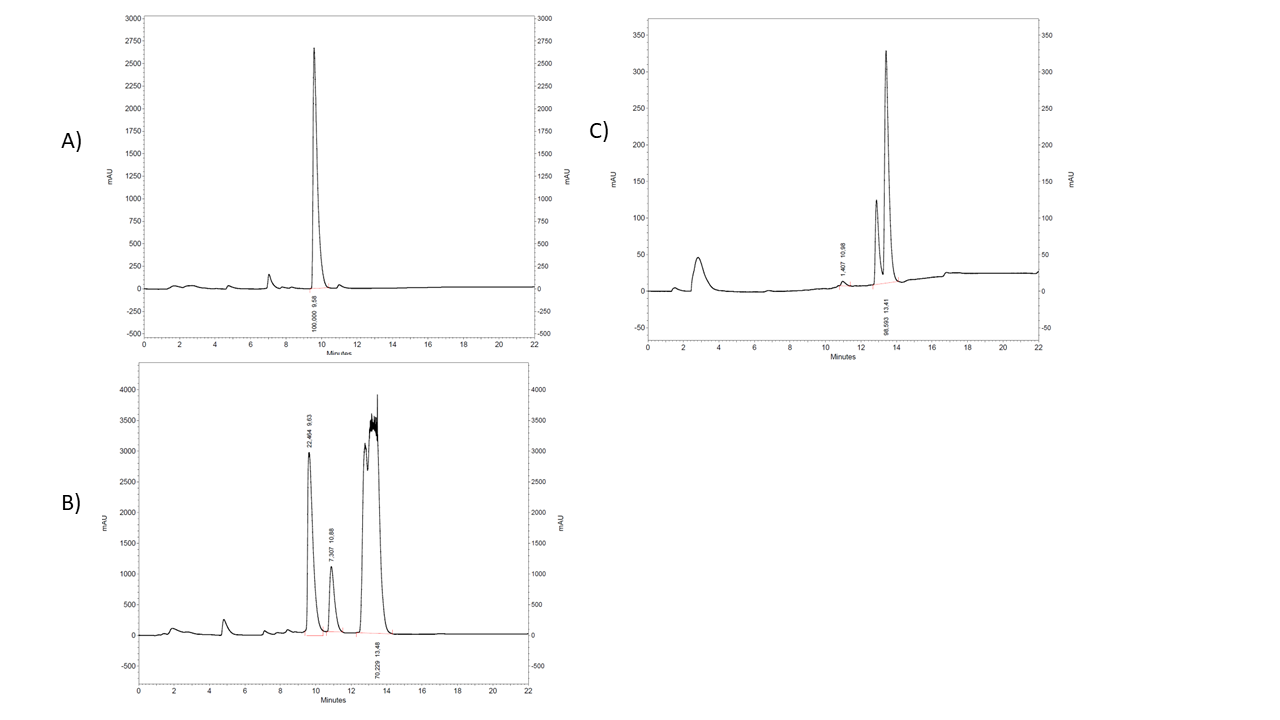
Figure S3.1.** UV chromatograms of A) Free H_2_L^diethyl^; B) reaction mixture of H_2_L^diethyl^, [AuCl(THT)] and Ga(NO_3_)_3_ C) isolated non-radioactive complex [Ga{Au(L^diethyl^)}_2_]^+^.


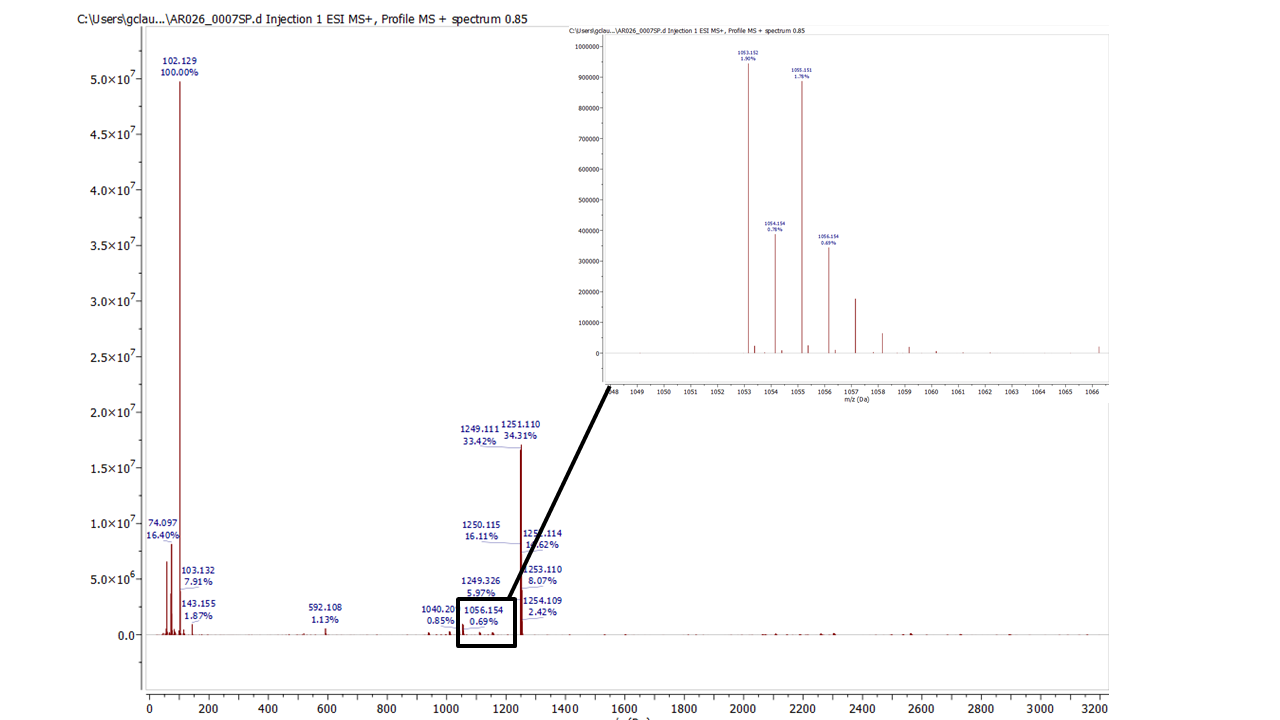


**Figure S3.2** ESI^+^ MS of [Ga{Au(L^diethyl^)}_2_] of the HPLC fraction at t_R_ = 9.8 min in water/acetonitrile.

**
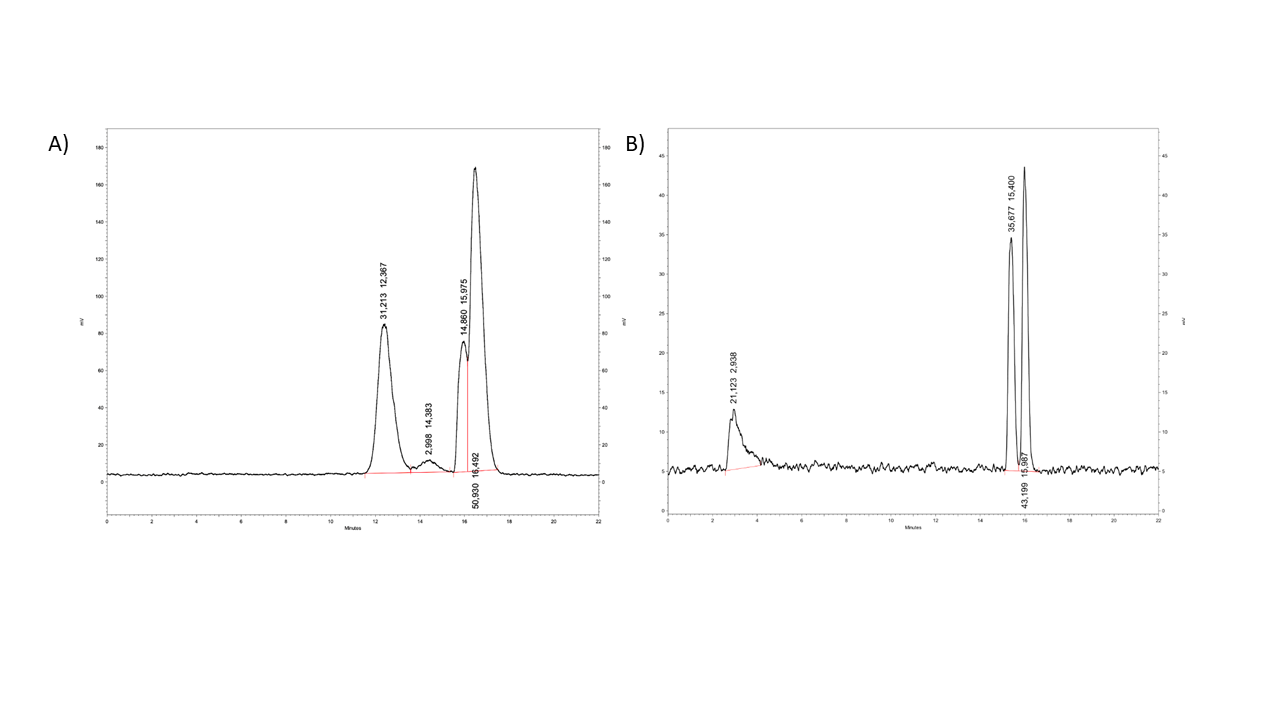
**

**Figure S3.3** Radio-chromatograms A) H_2_L^ethyl^, [AuCl(THT)] plus ^68^Ga in DMSO/Na-acetate B) starting from [Ga{Au(L^diethyl^)}_2_]^+^ and adding [^68^Ga]GaCl_3_; starting materials dissolved in DMSO; 90 °C 10 min.

*
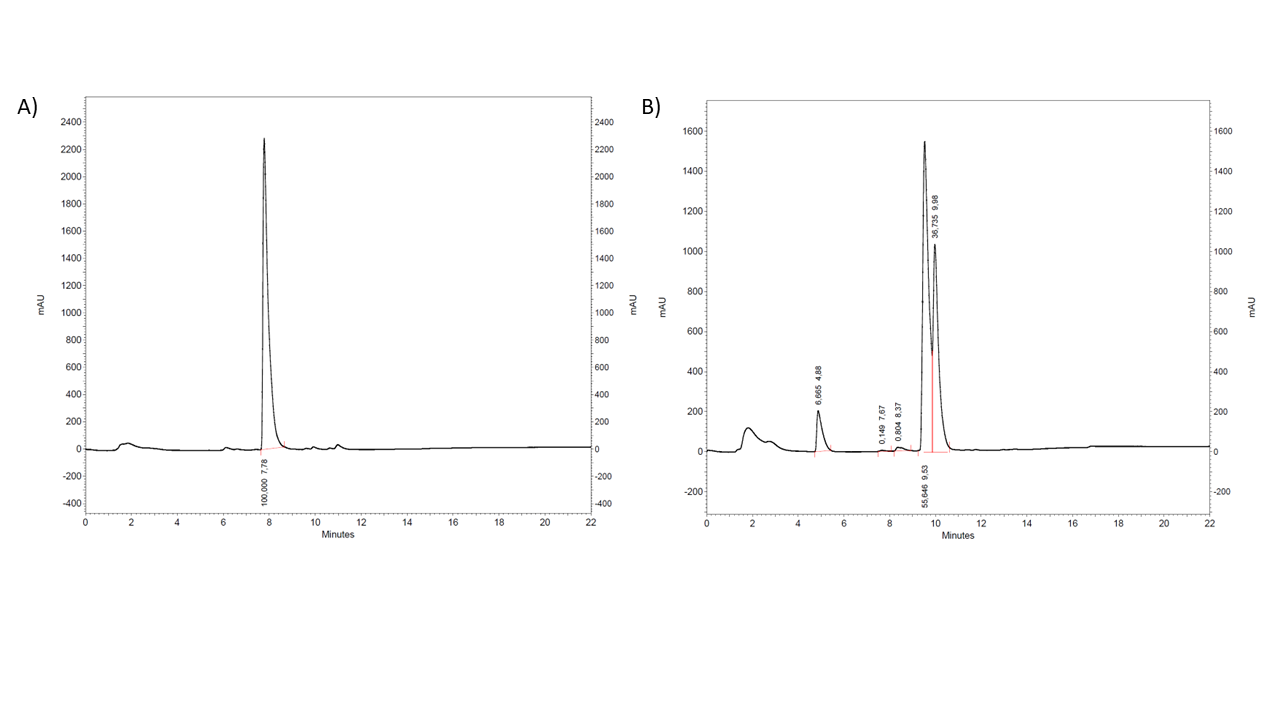
*

**Figure S3.4** UV Chromatograms A) Uncoordinated H_2_L^morph^ B) isolated non-radioactive complex [Ga{Au(L^morph^)}_2_]^+^.


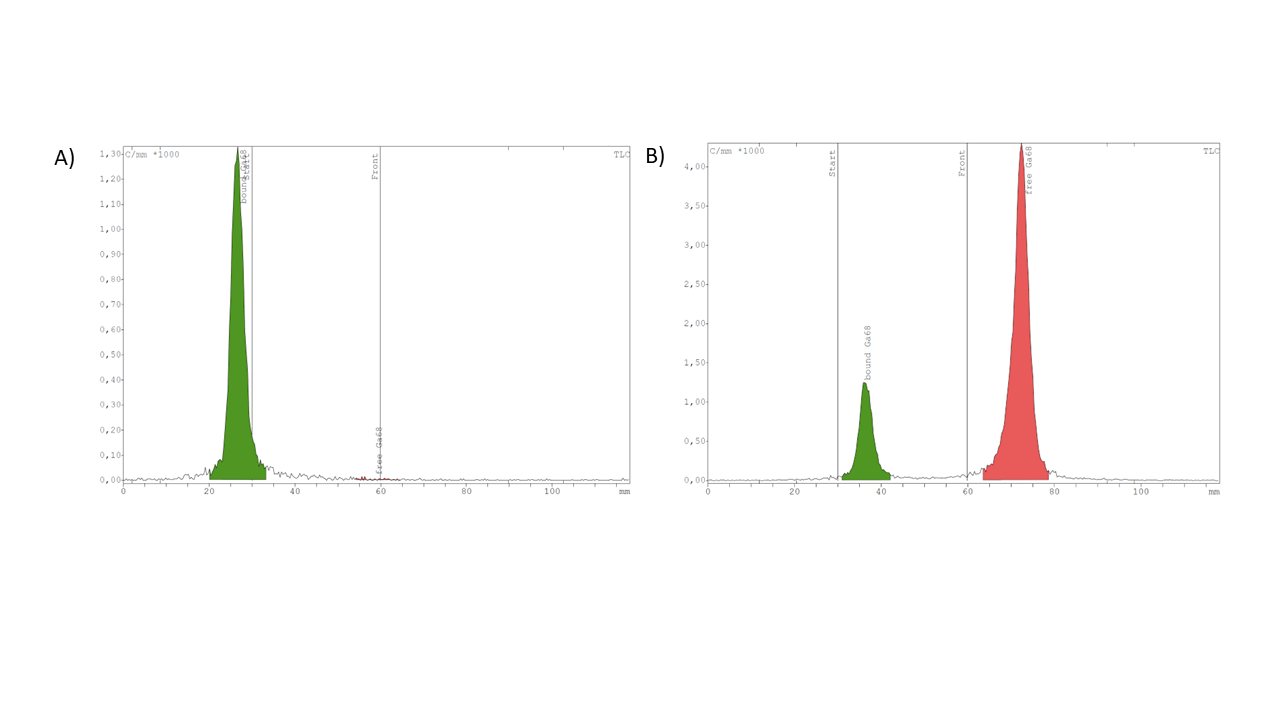


**Figure S3.5** iTLC-chromatograms of A) [^68^Ga][Ga{Au(L^morph^)}_2_]^+^ B) [^68^Ga][Ga{Au(L^morph^)}_2_]^+^ with human serum albumin.


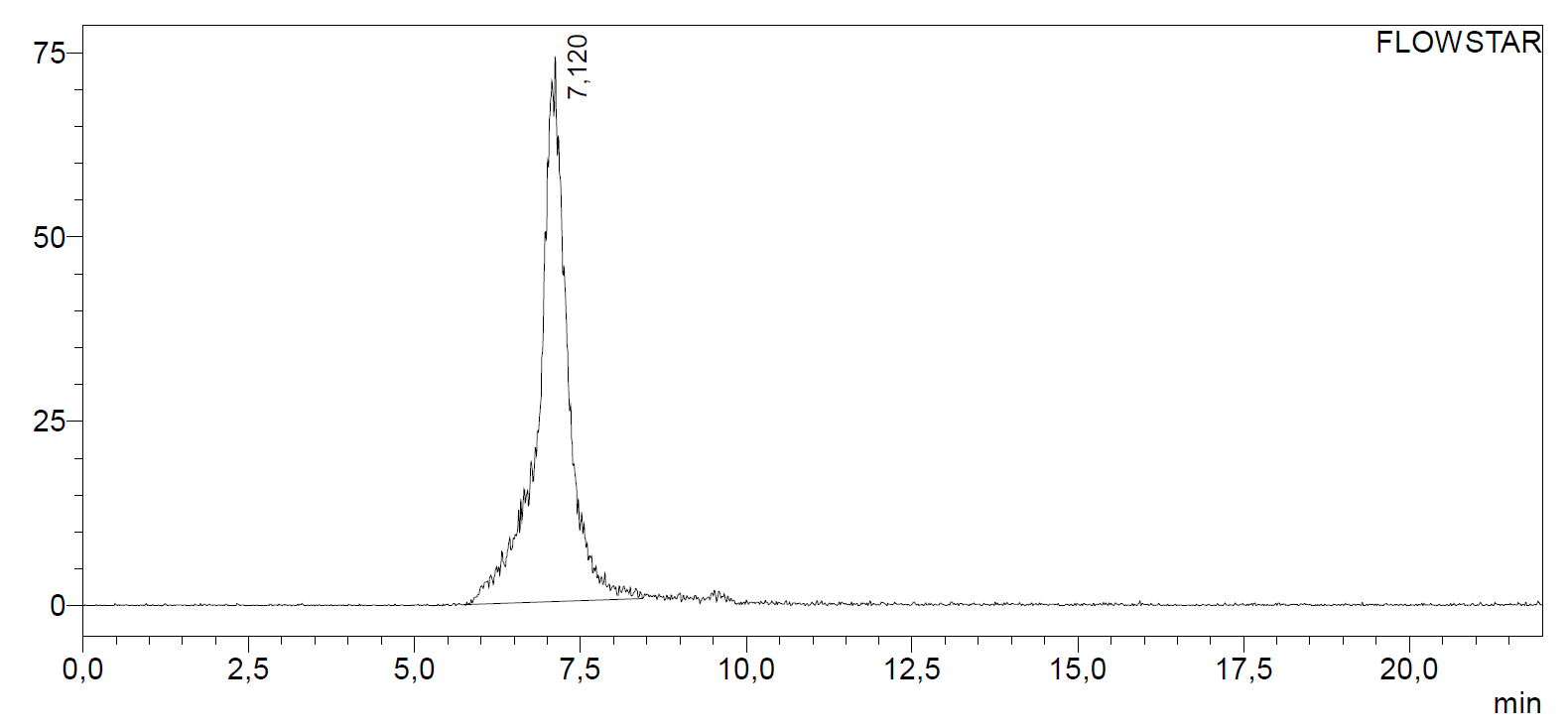


**Figure S3.6** Radio-Chromatogram of [^198^Au]AuCl(THT)].


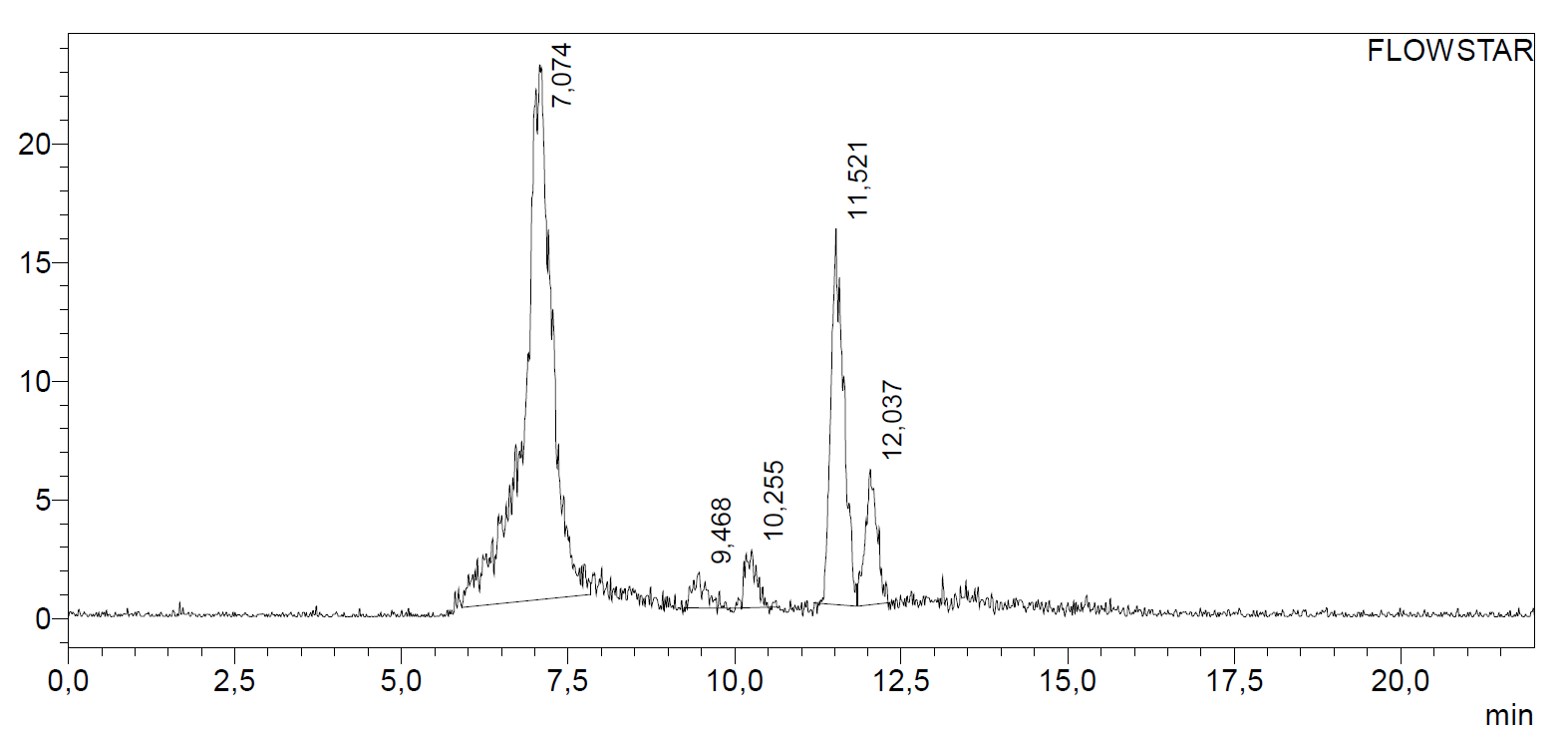


**Figure S3.7** Radio-Chromatogram of [^198^Au][Ga{Au(L^morph^)}_2_]^+^.


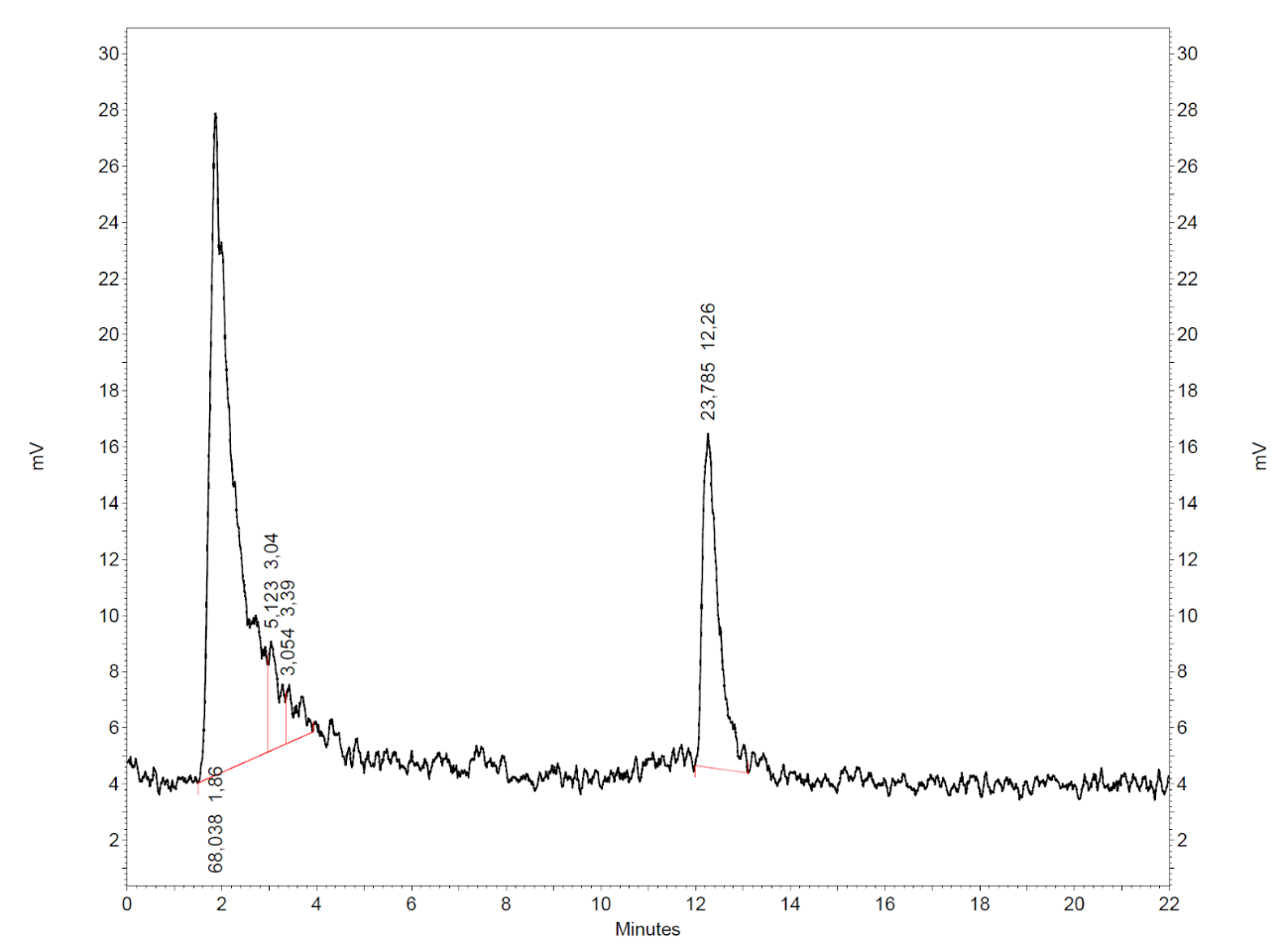


**Figure S3.8** Radio-Chromatogram of [^177^Lu][Lu{Au(L^morph^)}_3_].
